# Recycling of Waste Refractories from Failure Mechanisms to High-Value Regeneration Pathways: A Systematic Review

**DOI:** 10.3390/ma19214758

**Published:** 2026-11-09

**Authors:** Lingfeng Niu, Junjie Zhang, Mingyang Song, Hanlin Shen, Ming Yang, Bo Liu

**Affiliations:** 1 Institute for Advanced Materials and Technology, University of Science and Technology Beijing, Beijing 100083, China; 2 Shunde Innovation School, University of Science and Technology Beijing, Foshan 528000, China; 3 CRRC Tangshan Co., Ltd., Tangshan 064000, China; 4 State Key Laboratory of Advanced Metallurgy, University of Science and Technology Beijing, Beijing 100083, China; 5 Jianlong Beiman Special Steel Co., Ltd., Qiqihar 161041, China

**Keywords:** waste refractories, resource utilization, circular economy, failure mechanisms, recycling technologies

## Abstract

The high-speed growth of high-temperature industries has resulted in an increase in the generation of waste refractories (WRs) whose resource consumption is a significant concern towards low-carbon targets and the circular economy. This review thoroughly analyzes the mechanisms of physical failure, such as thermal shock and mechanical stress, and chemical failure, including oxidation, erosion, and permeation. Such processes cause compositional complexity and structural degradation, which are the main barriers to recycling. The review discusses the classification-based recycling approaches, including closed-loop recycling, which reprocesses waste into shaped or unshaped refractories through particle-size optimization and binder modification, and open-loop recycling, which converts waste into metallurgical or chemical feedstocks, construction materials, and functional ceramics or thermal storage media. Combining failure mechanisms with regeneration pathways, this review proposes innovative strategies for high-value recycling, offering systematic direction to the green transformation and sustainable use of resources in high-temperature industries.

## 1. Introduction

Due to their exceptional thermal shock resistance and physical and chemical stability, refractories have become indispensable functional materials in high-temperature industrial sectors such as iron and steel metallurgy, cement production, non-ferrous metallurgy, glass manufacturing, and petrochemical processing [1,2,3]. With the continuous growth of global industrial capacity and accelerated upgrades of thermal processing equipment, the demand for and consumption of refractories have steadily increased. Because of their relatively short service life, substantial quantities of waste refractories (WRs) are generated annually [4].

**Figure 1:**
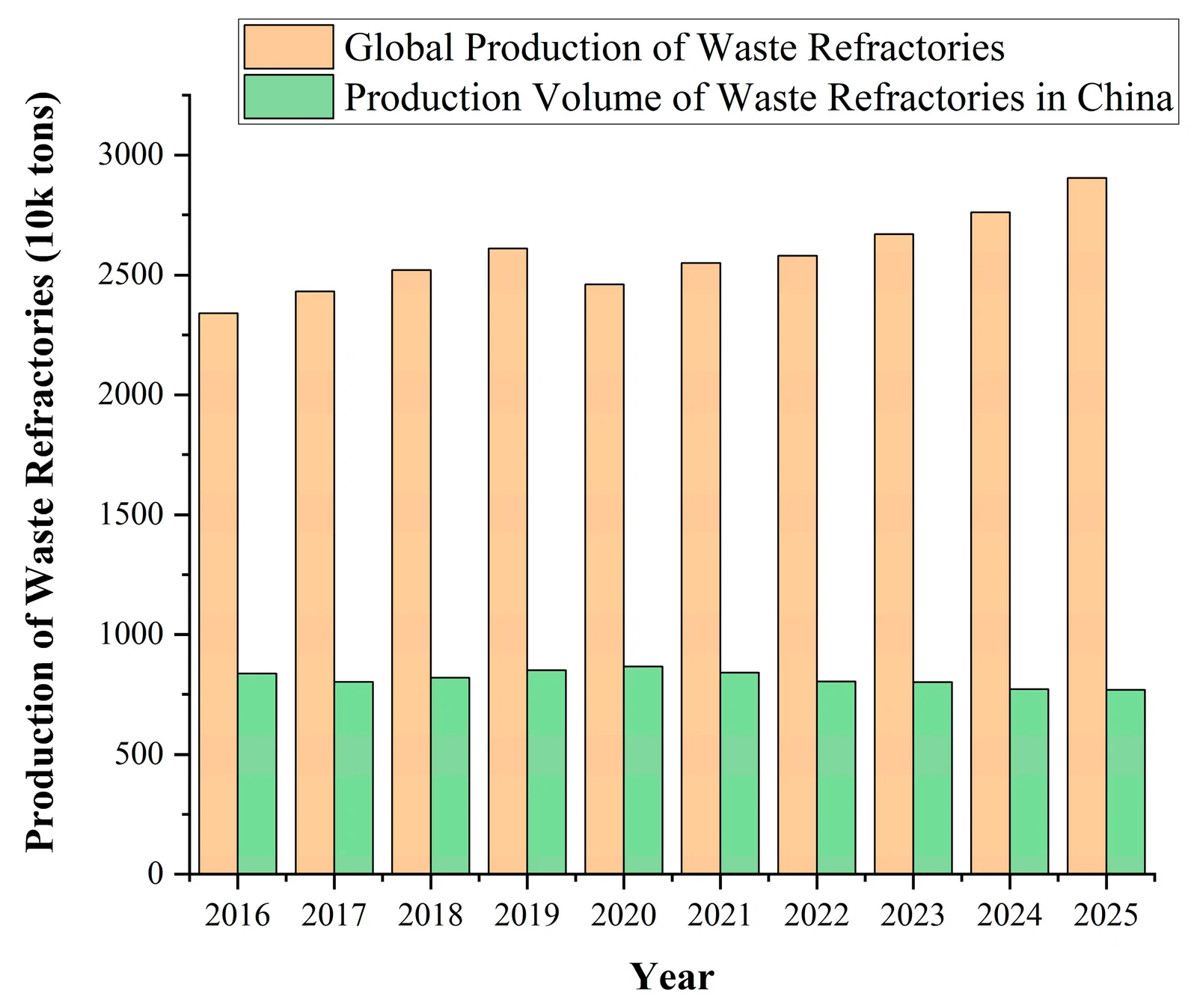
Comparison of waste-refractory generation quantity between China and the global total (2016–2025) [5,6,7,8].

Approximately 28 million tons of waste refractories are produced globally each year [5]. As the world’s largest producer and consumer of refractories, China accounts for about 30% of this global WR volume. Figure 1 illustrates the comparative trends in global and Chinese WR generation from 2016 to 2025 [5,6,7,8]. For 2020–2025, global waste-refractory generation was estimated by multiplying the corresponding refractory-consumption values by 0.65, representing the midpoint of the 60–70% fraction remaining after the reported 30–40 wt.% in-service consumption [5]. The Chinese estimates were calculated by multiplying the annual refractory production data by a constant factor of 0.35. The underlying production data for 2016–2018, 2019–2023, and 2024–2025 were obtained from Zeng [6,7,8], respectively. The same factor of 0.35 was consistently applied to the production data from all three sources. These values are indicative estimates rather than directly measured annual waste-generation statistics. It can be observed that global waste volumes fluctuate at a high level between 23 and 29 million tons, while China’s annual waste remains stable between 6 and 9 million tons. However, the overall recycling rate of WR in China is estimated at 30%, while Japan, South Korea, and some European countries have achieved recycling rates of 70–90% [5].

**Figure 2:**
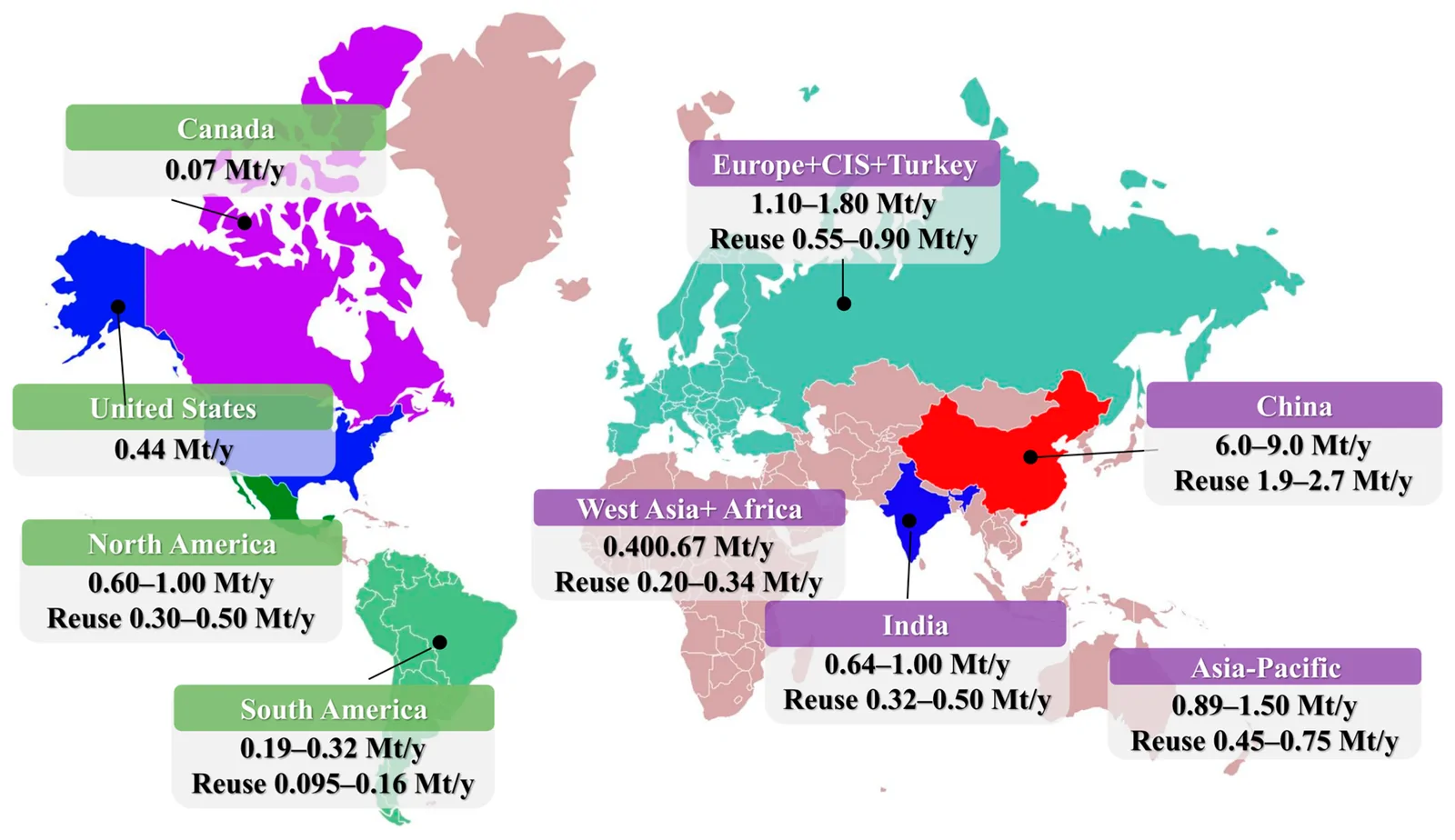
Estimated yearly waste refractory generation.

Figure 2 complements Figure 1 by showing the regional ranges of the global total data. The combined output of steel ladles and cement rotary kilns in Europe generates 403 kt of WR, of which 77.5 kt is recycled into new refractory production. Broader industry estimates are as follows: EUR, CIS and Turkey 1.11.8 Mt; North America 0.6–1.0 Mt; South America 0.19–0.32 Mt; China 6.0–9.0 Mt; India 0.64–1.0 Mt; Asia Pacific 0.89–1.5 Mt; West Asia and Africa 0.40–0.67 Mt [9,10]. This source also states that under current dismantling and processing conditions, approximately 50% of the WR can be reused in refractory production.

WRs typically consist of MgO, Al_2_O_3_, CaO, SiO_2_, and spinel-related phases, alongside impurities such as Fe_2_O_3_, alkali oxides, and residual slag components introduced during high-temperature service [4,5]. WRs exhibit high melting points, dense microstructures, and excellent chemical stability, which make them resistant to environmental degradation. Traditional disposal methods like landfilling and downgraded utilization not only occupy valuable land resources but also pose long-term environmental risks, including dust emissions and potential leaching of harmful elements [11]. Against the backdrop of global transitions toward carbon neutrality and resource-efficient manufacturing, the sustainable management and value-added processing of WRs are gaining increasing attention.

Compared with the mining and processing of primary mineral resources, recycling WRs significantly reduces energy consumption, greenhouse gas emissions, and dependence on non-renewable raw materials [12,13]. Consequently, the circular utilization of discarded refractories aligns closely with principles of the circular economy, industrial symbiosis, and globally promoted low-carbon manufacturing systems.

To date, recycling strategies for WRs can be broadly categorized into four types based on processing depth. The first approach is direct utilization, which involves the reuse of suitable refractory materials with little or no additional processing [14]. For example, Muñoz et al. [15] reported the direct reuse of spent MgO-C refractory bricks in a Spanish steelworks. Second is the most widely applied primary utilization method, where sorted, crushed, and screened materials are blended into new products. Mangngi et al. [16] investigated the use of recycled refractory brick aggregates as partial replacements for natural coarse aggregates in concrete. Similarly, Pertiwi et al. [17] demonstrated the utilization of waste refractory brick aggregates as substitutes for natural sand in concrete paving blocks. To enhance recycled material quality, intermediate utilization methods further incorporate chemical purification or specialized processing to approximate primary material properties. The most advanced approach is high-level utilization, achieving high-value resource utilization through composition modification and cross-domain conversion [14].

Despite significant progress, recycling WRs still faces challenges, including compositional heterogeneity, impurity accumulation, energy-intensive processing, and the lack of standardized evaluation criteria for recycled products [11,18]. Therefore, systematically understanding recycling pathways, material transformation mechanisms, and application scenarios is crucial for advancing the utilization of WR from low-grade recycling toward high-value, functional, and closed-loop material cycles.

To promote the sustainable development of the steel and high-temperature industries, it is urgently necessary to achieve the efficient, economical, and clean recycling of WRs. Hence, this review first analyzes the fundamental causes of refractory failure during service and then summarizes WR recycling methods, including closed-loop regeneration into refractory products and open-loop conversion into other useful materials.

## 2. Review Methodology

The recycling of WR materials involves multiple aspects, including in-service failure mechanisms, recycling technology pathways, and the application of recycled materials. The current research is widely distributed across various fields such as materials science, metallurgical engineering, chemistry and circular economy. Furthermore, individual studies typically reflect only specific material systems, recycling processes, or application scenarios. Therefore, in order to enhance the comprehensiveness of the review, this study adopts a systematic literature review approach to develop a systematic understanding of WR materials.

The literature search and study selection process followed a workflow based on the Preferred Reporting Items for Systematic Reviews and Meta-Analyses (PRISMA) guidelines. This framework emphasizes the need to clearly define the scope of the search, systematically document the database search process, remove duplicate records, ensure transparency in the selection process, assess full-text compliance, and report the studies ultimately included in the analysis [19].

**Figure 3:**
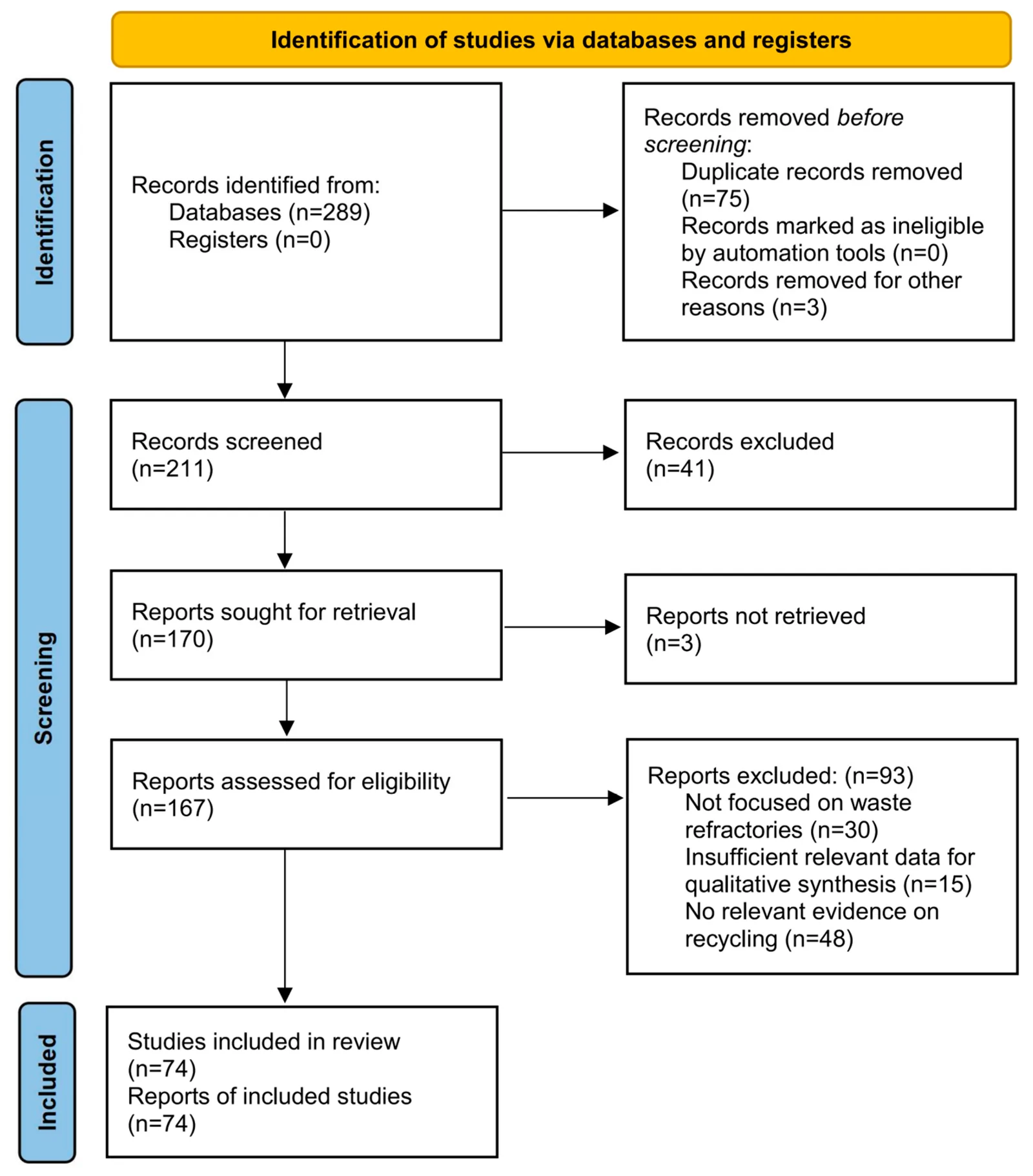
PRISMA 2020 flow diagram of the literature selection process.

In this study, the scope of the review is limited to research on the characterization, treatment, recovery, and reuse of waste refractories. Relevant literature was retrieved from Web of Science and Scopus, covering the period from 2015 to 2026. Selected studies published before 2015 were additionally cited to provide historical context and illustrate early developments in refractory recycling. These earlier studies were not included in the formal study selection, bibliometric analysis, or systematic comparative assessment. The database-specific Boolean strings were as follows: Web of Science Core Collection, TS= ((“waste refractory” OR “waste refractories” OR “spent refractory” OR “spent refractories”) AND (recycle OR recover OR recovery OR reuse OR regeneration OR valorization)); Scopus, TITLE-ABS-KEY ((“waste refractory” OR “waste refractories” OR “spent refractory” OR “spent refractories”) AND (recycle OR recover OR recovery OR reuse OR regeneration OR valorization)). All searches were conducted on 21 June 2026. A total of 289 records were initially identified, including 203 from Web of Science and 86 from Scopus. After 75 duplicate records and three records unrelated to the processing and recycling of WRs were excluded, the remaining 211 records entered the screening stage. Subsequently, the full texts of 170 potentially relevant studies were evaluated based on predefined screening criteria, and 74 studies were ultimately included in this review. The complete research screening process is presented graphically as shown in Figure 3.

To elucidate the relationships among the research topics, a keyword co-occurrence analysis was conducted using VOS viewer version 1.6.21. The input dataset consisted of 203 records retrieved from Web of Science and was used to illustrate the broader research topics and keyword associations related to waste refractories. The literature records were obtained from Web of Science and exported as text files. The analysis was based on both titles and keywords. Prior to analysis, keywords were standardized by merging synonyms, singular and plural forms, and abbreviations. The minimum occurrence threshold was set at five, resulting in a network containing 51 keywords. Keyword co-occurrence was analyzed using full counting, and the network was normalized using association strength.

**Figure 4:**
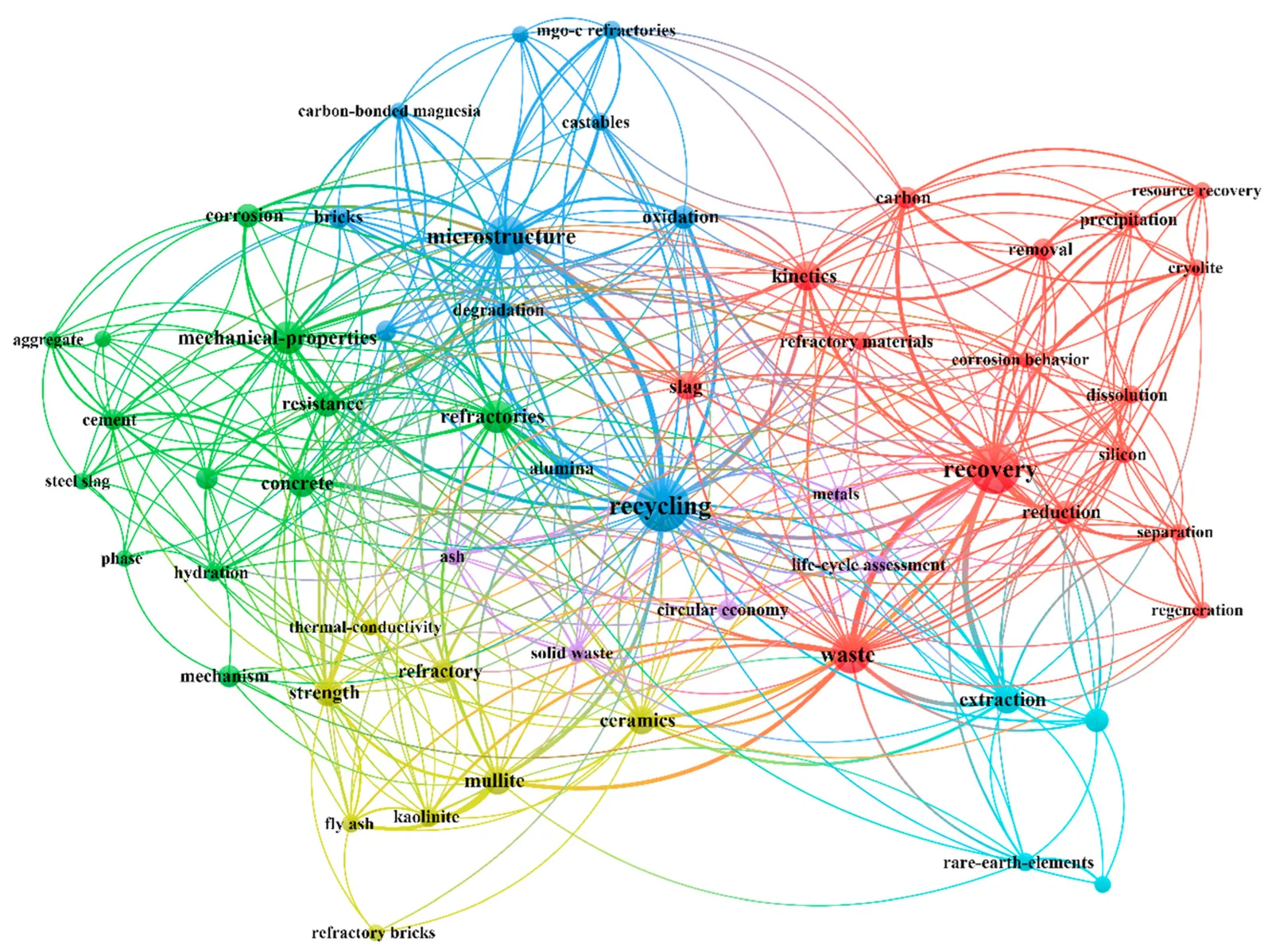
Keyword co-occurrence network map of research on waste refractories.

Figure 4 shows a co-occurrence analysis of keywords in the literature regarding the recycling of WRs. The bibliometric review shows that the concept of WR recycling has developed over time to become more than just a simple reuse of materials but rather various functional oriented approaches.

## 3. Failure Mechanisms of Refractories

Refractories can withstand high temperatures and retain their mechanical properties when exposed to corrosive liquids or gases. Understanding the failure mechanisms of refractories is crucial for addressing the disposal of WR.

### 3.1. Physical Failure

Intense thermal and mechanical conditions during the service of refractories in high-temperature kilns lead to physical failure. Refractories are an integral part of sustaining a thermal environment inside these kilns. This operation leads to the creation of large temperature gradients in the material. The surface layer directly contacting the heat source is much hotter than the backing layer. The resultant thermal gradient produces unequal expansion of different material parts, which causes thermal stresses. Microcracks are initiated and propagated when the mechanical performance limit of the material is exceeded, ultimately causing structural damage.

The physical failure of refractories in high-temperature service environments usually occurs in three typical forms.

Thermal shock: Due to abrupt changes in kiln temperature, significant thermal gradients develop in the material, which leads to uneven thermal expansion of different sections and internal stresses [20]. When the stresses are greater than the material strength limit, microcracks grow and spread over each other, forming a network of through cracks.

Mechanical stress: In operation, refractories are exposed to a variety of mechanical loads, such as impact forces caused by materials, static pressure caused by the masonry structure, scouring forces caused by gas flow or molten materials [21]. Working surface fragmentation, spalling, and wear are caused by external forces that exceed the material mechanical strength.

Structural evolution: Refractories experience additional sintering and densification, which results in reduced volume and increased brittleness. As the temperature reaches the refractoriness of the refractories, plastic deformation processes like softening and creep may take place. The high-temperature effects alter both microstructure and macroscopic properties of the refractories, leading to more rapid failure.

All three types of physical failure contribute to weakening refractories, which leads to gradual degradation of their original intact form into a complicated system with numerous cracks and non-uniform fragmentation. This not only reduces their service life but also poses significant challenges for subsequent recycling and reuse.

### 3.2. Chemical Failure

Chemical failure involves irreversible reactions between refractories and their service environment, including furnace slag, molten metal, dust, and exhaust gases. The final result is a radical change in the material composition, phase and structure, which results in serious impediments to recycling. These are some of the chemical failure reactions that may occur in refractories during service.

(1) Oxidation reactions

The components in the refractories may undergo oxidation reactions. As an illustration, carbon-containing refractories (carbon bricks or silicon carbide bricks) can react with O_2_ to produce CO or CO_2_ (Equations (1) and (2)). In carbon-containing materials, carbon consumption and the release of gaseous products can increase porosity, reduce bulk density, and weaken the refractory structure. These changes may compromise mechanical integrity and increase material losses during subsequent crushing, sorting, and recycling. The effects of SiC oxidation on the microstructure and performance, however, depend on whether the resulting silica-rich layer provides protection or contributes to further degradation.

C + O_2_ → CO_2_ (1)

SiC + 2O_2_ → SiO_2_ + CO_2_ (2)

(2) Corrosion reactions

When refractories come into contact with molten metal or slag, corrosion reactions may occur. These reactions typically happen at high temperatures, where components within the refractory react with the molten material to form new phases or dissolve into the melt. This renders mechanical separation nearly ineffective and significantly increases the difficulty of purification and homogenization. For example, reactions between alumina–carbon refractories and iron-containing species in molten steel can cause corrosion and structural damage (Equations (3) and (4)).

(3) $${{\mathrm{Al}}_{2}O}_{3}+\mathrm{FeO}\to {{\mathrm{FeAl}}_{2}O}_{4}$$

SiO_2_ + CaO → CaSiO_3_ (4)

(3) Permeation and diffusion reactions

Elements from molten slags or metals may infiltrate the pore network of refractories and diffuse into their constituent phases. For example, iron-bearing species in a slag may enter a magnesium aluminate spinel-containing refractory and alter its local phase composition. Under suitable chemical conditions, FeO may react with MgAl_2_O_4_ to form FeAl_2_O_4_ (hercynite) and MgO (Equation (5)). Such compositional changes may modify the local microstructure and thermomechanical properties of the refractory. The extent of this transformation depends on the slag composition, temperature, and oxygen potential.

(5) $${{\mathrm{MgAl}}_{2}O}_{4}+\mathrm{FeO}\to {{\mathrm{FeAl}}_{2}O}_{4}+\mathrm{MgO}$$

(4) Carbonization and nitriding reactions

Under suitable high-temperature and reducing conditions, oxide constituents in refractories may undergo carbothermal reduction or carbothermal reduction nitridation. For example, silica may react with carbon to form silicon carbide, whereas alumina may react with carbon in the presence of nitrogen to form aluminum nitride. These reactions require appropriate temperature and gas-composition conditions and should not be assumed to occur in all refractory systems. When they occur, the formation of carbide or nitride phases may alter the phase assemblage, pore structure, and mechanical properties of the material (Equations (6) and (7)).

SiO_2_ + 3C → SiC + 2CO (6)

(7) $${{\mathrm{Al}}_{2}O}_{3}+N_{2}+3C\to 2\mathrm{AlN}+3\mathrm{CO}$$

(5) Reduction reactions

Under reducing atmospheres, oxides within refractories may be reduced to metals or other lower-valent oxides. For example, iron oxide may be reduced to metallic iron, leading to phase composition and structural stability changes (Equation (8)).

(8) $${{\mathrm{Fe}}_{2}O}_{3}+3C\to 2\mathrm{Fe}+3\mathrm{CO}$$

(6) Crystallization temperature and melting point reduction

Refractories undergo crystalline phase transformations at high temperatures or react with molten substances to form low-melting-point compounds, reducing their molten corrosion resistance. For example, silicate refractories react with alkali metal oxides to form low-melting-point silicate compounds (Equation (9)).

2Na_2_O + SiO_2_ → Na_4_SiO_4_ (9)

Sodium-containing substances can cause substantial degradation of high-chromia refractories. The severity order of liquid-phase corrosion is L-NaCl > L-NaOH > L-Na_2_SO_4_ > L-Na_2_CO_3_ > L-NaAc, while the impact order of gas-phase corrosion is G-Na_2_CO_3_ > G-Na_2_SO_4_ > G-NaOH > G-NaAc > G-NaCl. It is worth noting that organic sodium (NaAc) significantly contributes to the formation of protective spinel layers, while water-soluble sodium (especially Na_2_CO_3_ and Na_2_SO_4_) causes deep penetration and corrosion of refractories [22].

Zhou et al. [23] studied the degradation mechanism of the magnesia–magnesium aluminate used in the transition zone of cement rotary kilns and discovered how the combined effects of physical and chemical failures worsen recycling difficulties. Their analysis indicates that the permeation of alkaline components (K_2_O, Cl) initiates chemical erosion, forming compounds such as potassium chloride (KCl) within the brick structure. More critically, the study revealed how chemical erosion and physical stress mutually aggravate, ultimately leading to structural failure: on one hand, infiltrated alkaline components react with cement clinker and spinel phases to form liquid phases, causing densification of the reaction layer and altering its thermophysical properties; on the other hand, under the alternating thermal field induced by kiln rotation, these chemical erosion products interact with thermomechanical stresses, dramatically accelerating crack propagation and ultimately triggering structural spalling. Gao et al. [24] discovered through numerical simulation analysis that the degradation of high-chromium refractory bricks is mainly due to structural peeling induced by the slag corrosion and thermal stress produced by temperature gradients. The stress cracks generated at the corrosion interface provide a permeation path for the slag and act as a subsurface for further corrosion, resulting in additional corrosion and peeling. Phosphate addition promoted the formation of a CaO-Na_2_O-P_2_O_5_-rich liquid phase, increasing slag viscosity and improving the resistance of refractories to infiltration by low-Na_2_O slags (≤10%) [25].

The reaction between slag and refractories can be divided into an infiltration zone and a reaction zone. The Fe and Mg in the slag diffuse into the reaction layer of the refractories. With the capillary infiltration of molten slag, the amount of Si, Ca, and Al increases, which increases the corrosion area and forms a permeable layer. However, a denser structure can be observed in the reaction layer than in the permeable layer, mainly due to the reaction between the molten slag and the original refractory material [26].

Belgacem et al. [27] believe that testing WRs can provide crucial insights for predicting refractory failure behavior. They studied refractories from a cement rotary kiln with a processing capacity of 4000 t/day that had been in operation for 40 days. Samples were collected from the heated zone located 3.6 to 15 m from the kiln exterior, with lengths of 125.2 mm and 84.2 mm, respectively. Their study revealed that used refractories exhibited chemical composition variations across different regions compared with new bricks. Used bricks showed slight increases in CaO and SiO_2_ content, with trace amounts of sulfur and potassium present on the heated surface and the interface exposed to high-temperature cement clinker. The refractory zones in direct contact with the cement clinker melt exhibited entirely different phases compared with the original bricks (mainly MgAl_2_O_4_). They were phases formed by binary systems (CaO-Al_2_O_3_), ternary systems (CaO-Al_2_O_3_-SiO_2_), and CaO-MgO-SiO_2_, mostly made up of calcium aluminate.

Refractory corrosion by “liquid” silicates was closely correlated with variations in composition, viscosity, time, temperature, and system atmosphere [27]. The presence of molten salts and mineralized cations enhances fluidity while simultaneously influencing reaction kinetics and stability by generating eutectic phase points. This leads to the phenomenon of liquid phase permeating through the open pores of refractories. Under these conditions, corrosive liquid phases containing calcium, aluminum, and silicon can permeate into refractory bricks, leading to their chemical failure. The corrosion reaction of magnesium aluminum spinel primarily occurs between 1000 and 1350 °C, as detailed in the following reaction [24]:

MgAl_2_O_4_ + CaO · SiO_2_ → CaAl_2_O_4_ + MgSiO_3_ (10)

### 3.3. The Resulting Challenges for Recycling Technology

Physical and chemical failure mechanisms not only shorten the service life of refractories but also pose substantial challenges to WR recycling. Physical failure creates the primary obstacle to recycling by disrupting the macrostructure of materials. Thermal shock-induced crack networks, mechanical stress-induced fragmentation and spalling, and structural deformation result in inconsistent particle size and severe pulverization of WR [28]. This not only renders automated sorting inefficient but also means recycled materials inherit structural defects, leading to significant degradation of bulk density and mechanical strength. Moreover, cracks and voids facilitate impurity penetration, hindering subsequent processing.

Chemical failure essentially changes the natural chemical and mineralogical structure of the refractories and creates a stronger technical barrier to the high-value utilization [29]. Interactions between refractories and slag or metal vapors can form low-melting-point phases and cause internal contamination. These impurities are strongly bound by chemical bonding, so that they cannot be separated using physical methods and thus energy-intensive melting or expensive chemical leaching is required to purify them. Furthermore, the introduction of toxic elements (e.g., Zn, Cr and Cu) decreases the economic viability and environmental safety of the recycling process.

Physical and chemical damage interact during service, and their combined effects can exceed those of either mechanism acting independently. Cracks offer very efficient channels to allow quick penetration of slag and other impurities, greatly increasing the area of chemical reaction interface. At the same time, new phases produced by chemical corrosion have dissimilar thermal expansion coefficients, which produce local stresses and promote the growth of microcracks.

**Figure 5:**
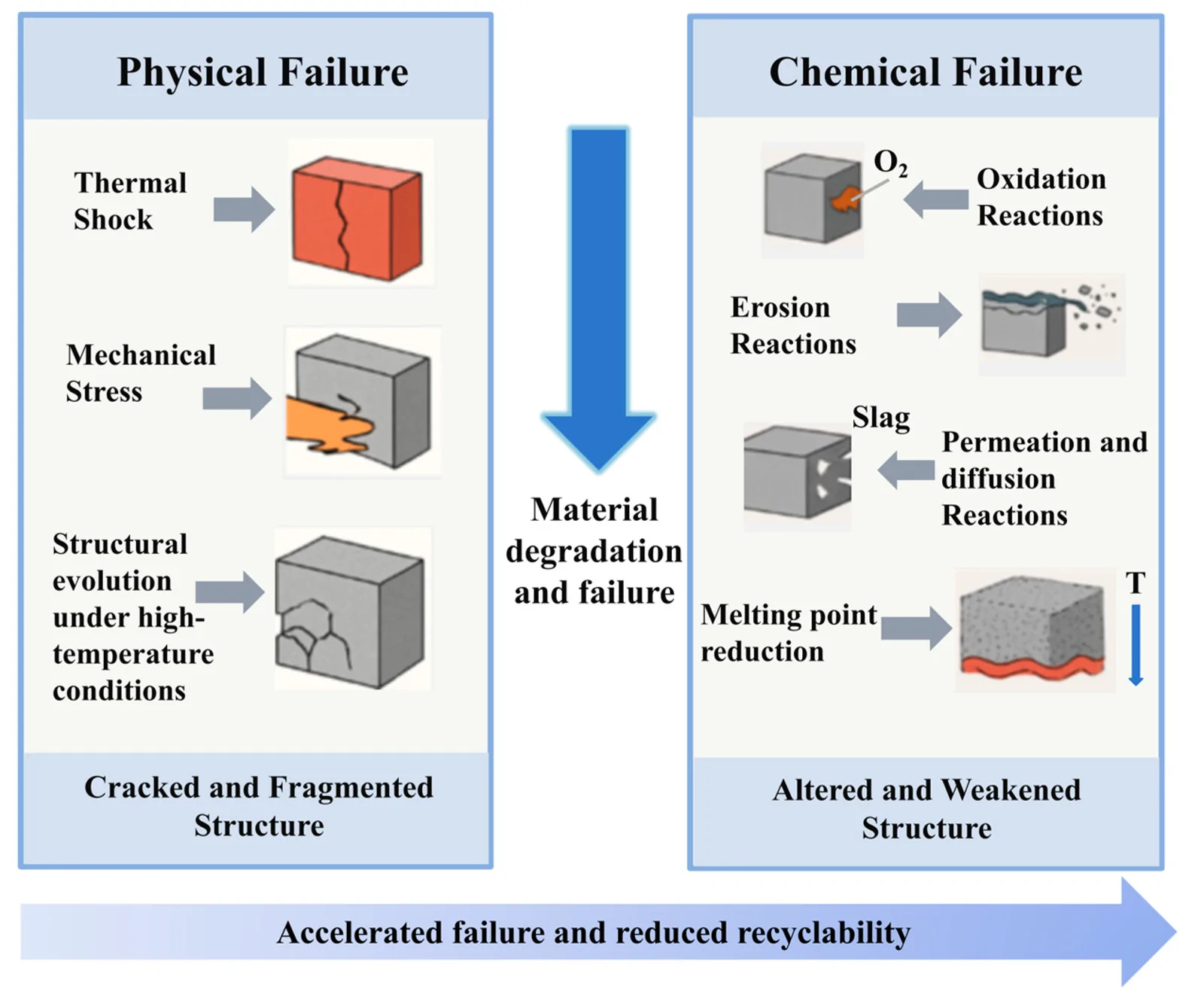
Classification of failure mechanisms in refractories.

Figure 5 presents a classification diagram of failure mechanisms for refractories to illustrate their physical and chemical failure pathways.

The selection of a recycling strategy should consider the relationship between degradation mechanisms and the characteristics of WRs. Physical damage caused by thermal shock and mechanical stress mainly leads to cracking and fragmentation. When chemical contamination is limited, sorting, crushing, and particle-size classification can enable closed-loop recycling into shaped or unshaped refractories. In contrast, oxidation, slag corrosion, and penetration may introduce impurities and secondary phases, requiring the removal of contaminated layers, stabilization, or chemical treatment before refractory regeneration. When the treated materials still cannot meet the requirements for closed-loop recycling, open-loop recycling can be considered based on their remaining chemical and physical properties. For example, MgO-rich waste refractories can be used as slag-conditioning materials in steelmaking, while suitable recycled refractory aggregates can be incorporated into construction materials. Therefore, degradation characteristics and pretreatment requirements should be jointly considered when selecting appropriate closed-loop or open-loop recycling pathways.

## 4. Closed-Loop Recycling

Recycling WRs into shaped or unshaped refractories can replace raw materials without significant alteration in material properties, resembling a closed-loop recycling pathway. This closed-loop system maximizes economic returns and advances the circular economy.

As discussed in Section 3, the service-induced physicochemical degradation introduces structural defects and elevates impurity levels in WRs (Table 1). The rise in impurity components alters the Ca/Si and Mg/Al ratios, thereby changing the melting point of the refractories, which leads to reduced high-temperature resistance and durability.

Furthermore, recycled refractory aggregates typically exhibit higher porosity and lower density than raw materials. This increases the specific surface area available for reactions between slag, molten metal, and high-temperature gases, accelerating chemical degradation. Simultaneously, higher porosity introduces material defects, further compromising the physical and mechanical properties of the refractory. Therefore, the quantity of recycled refractory aggregates that can be incorporated into refractory mixtures is limited and largely depends on the physicochemical properties of the recycled fraction, such as purity and porosity. Consequently, appropriate sorting and mineral processing are required to treat waste refractories.

### 4.1. Regeneration of Shaped Refractories

As a prime example of recycling, the technology for reusing shaped refractories is the most mature, with its core principle being the processing of WR for reuse as aggregate or matrix.

#### 4.1.1. Carbon-Containing Refractories

Significant progress has been made in the recycling of spent MgO-C bricks. Ludwig et al. [30] crushed waste MgO-C bricks into two aggregate sizes (1–4 mm and 4–6 mm) to fabricate recycled bricks. Following heat treatment at 1000 °C and 1500 °C, the samples exhibited lower density and poorer mechanical properties because of resin volatilization, thermal expansion mismatch, and MgO reduction. The difficulty in recycling waste bricks containing antioxidants (such as aluminum) lies in the chemical instability of impurity phases. To address this challenge, they proposed an ingenious solution to replace traditional coal tar pitch binder with phenolic varnish. Phenolic varnish has no softening stage before pyrolysis. These properties help suppress the volumetric expansion associated with Al_4_C_3_ hydration and preserve the structural integrity of the recycled refractories.

**Figure 6:**
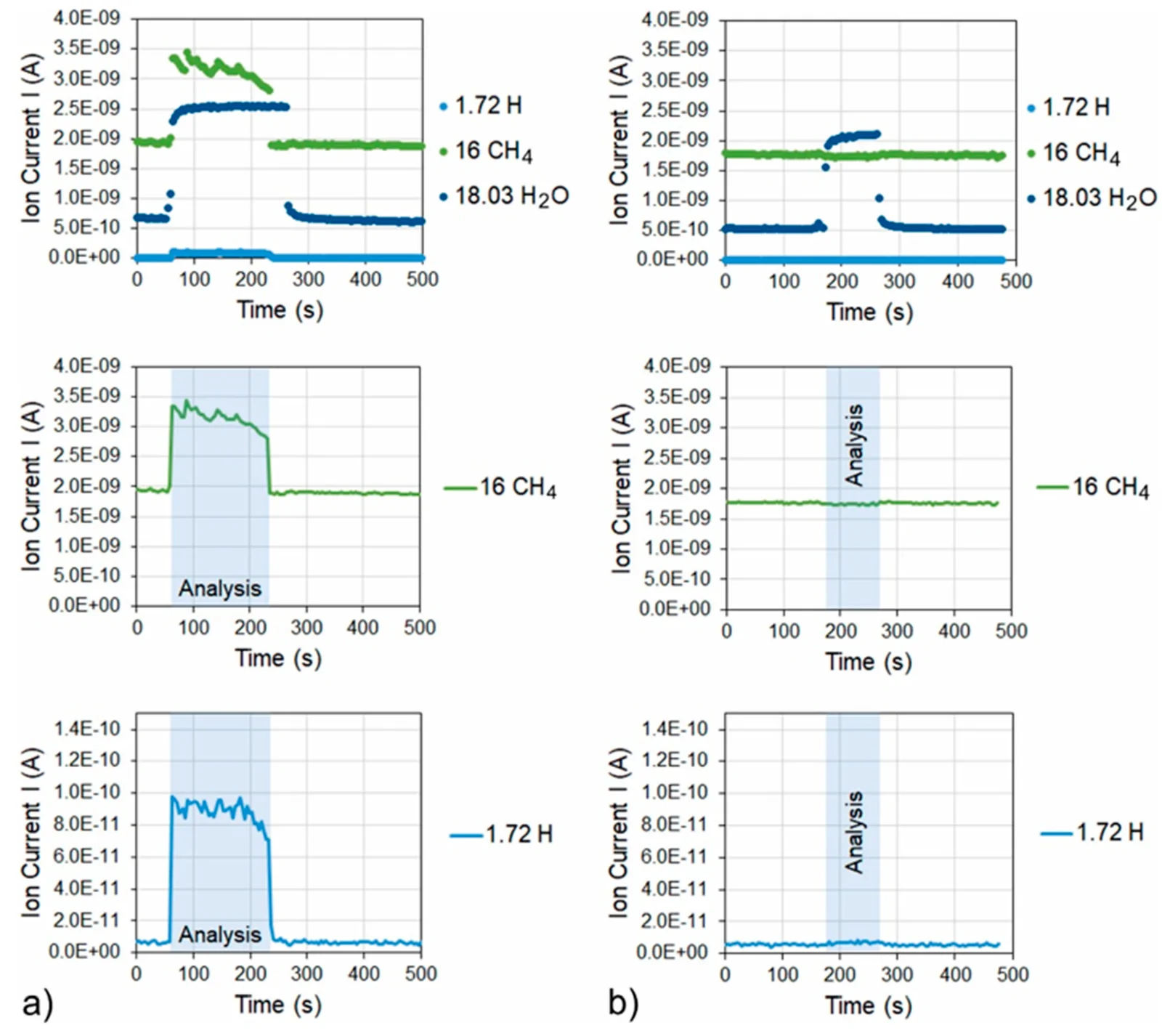
(**a**) Gases formed by the reaction of water with ground WR in the 0–1 mm particle size range and (**b**) gases produced by the reaction of water with a sample without WR [31].

Nevertheless, one of the major bottlenecks in recycling WR bricks with antioxidants (e.g., aluminum) is the chemical instability of these impurity phases. According to Moritz et al. [31], aluminum in the waste MgO-C is present as metallic Al and Al_4_C_3_. The latter reacts severely with moisture, exhibiting persistent gas evolution (CH_4_ and H_2_) in the WR (Figure 6), which causes serious volumetric expansion and cracking during thermal treatment. As a solution to this problem, an innovative approach to binding was suggested whereby traditional coal tar pitch was replaced with phenolic varnish. Phenolic varnish has a higher structural stiffness and completely lacks a softening stage prior to pyrolysis; it effectively suppresses the destructive volumetric expansion triggered by Al_4_C_3_ hydration, thereby safeguarding the structural integrity of the recycled refractories.

Apart from being directly recycled into similar refractory bricks, upcycling of spent MgO-C bricks is another potential valorization route that could be pursued. Chen et al. [32] utilized spent MgO-C bricks and Al_2_O_3_-MgO-C bricks as raw materials. After being ground to a particle size smaller than 45 μm, the materials were mixed with industrial alumina powder, followed by ball milling, drying, and a final sintering in a nitrogen atmosphere at 1723–1823 K. Refractory ceramics with magnesia–alumina spinel and MgAlON as the primary crystalline phases were successfully synthesized. Their study on the slag resistance mechanism revealed that slag erosion is jointly controlled by chemical reactions and diffusion, with an apparent activation energy of 274.6 kJ/mol. During erosion, elements such as Si, Ca, and Ti diffuse from the slag into the material, while Mg and Al migrate from the material into the slag.

Cheng et al. [33,34] further realized the in situ synthesis of MgAlON and its composites with MgO from spent MgO-C bricks via carbothermal reduction nitridation. Concerning the effect of impurity phases present in waste materials, it was found that impurities such as CaO and SiO_2_ finally remained in phases such as CaMgSiO_4_ and MgFe_2_O_4_. The results showed that both the MgO and C content introduced through the WR play a synergistic role in the flexural strength of the WR. They examined the wetting properties between the molten slag and the composite with the help of the drop-on-drop method [34]. Increasing the MgO content of the composite from 4.2 wt.% to 15.7 wt.% increased the contact angle with the slag to 91°, indicating reduced slag wettability.

**Figure 7:**
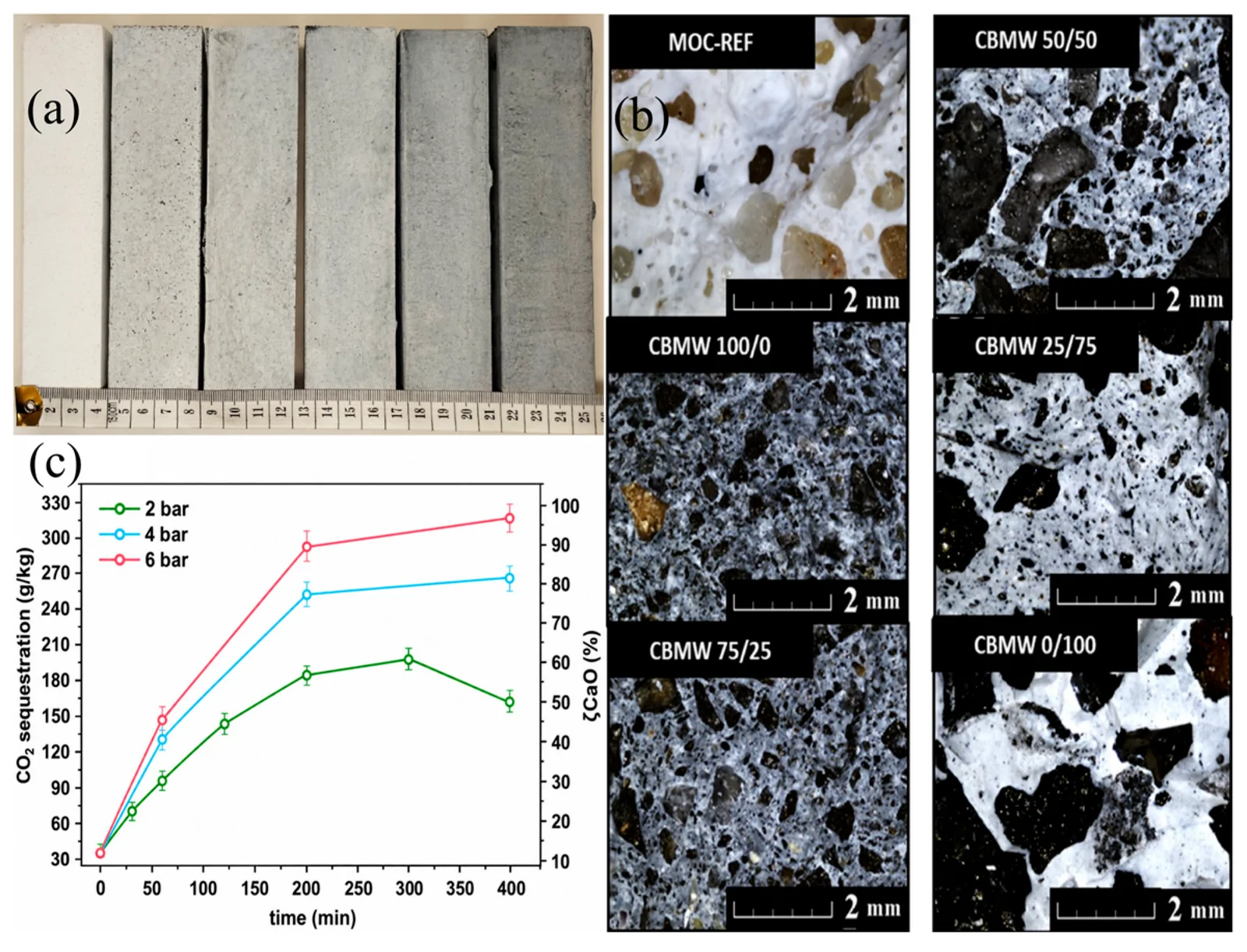
(**a**) Photographs of samples prepared by waste MgO-C-based refractories [35], (**b**) microstructure of magnesium oxide-based composite materials [35], (**c**) CO_2_ sequestration capacity at 15–390 min and 2–6 bar [36].

Spent MgO-C refractories were used as a green filler in the development of magnesium oxychloride cement-based composite materials [35]. To achieve optimal material properties, a binary particle size distribution was adopted to completely substitute traditional quartz sand. The obtained green eco-friendly cementitious composite was found to have a very high microstructural density with no defect and had a compressive strength of 78.1 MPa. An optical microscope (Figure 7b) was used to observe the microstructure of the fabricated magnesium oxychloride cement composite [35].

The study of carbon sequestration on solid waste has in recent years attracted continuous interest in the field of environmental management and sustainable development. Mineralization is an encouraging option to mitigate CO_2_ emissions as well as stabilize high-alkalinity waste, demonstrating the feasibility of utilizing industrial byproducts for CO_2_ capture and storage. Capelo-Avilés et al. [36] combined carbon sequestration with the recycling of carbon-containing WR by using three types of WRs as raw materials for CO_2_ mineralization. The WR exhibited a maximum carbonation degree of 79% and CO_2_ sequestration capacity of 165 g CO_2_/kg slag. Furthermore, they observed that CaO converted to CaCO_3_ during carbonation while MgO remained largely inert (Figure 7c) [36], and the process could effectively immobilize Pb.

Hou et al. [37] first stripped the altered layer from spent Al-Mg-C bricks and removed impurities. The particles obtained through crushing, air separation, and screening were coated with thermosetting phenolic resin and blended with Y_2_O_3_ and SiC additives to produce recycled refractory raw materials. Shen [38] processed uniform particles of WR < 10 mm and added compound additives during wet milling, followed by homogenization through a conditioning process. After undergoing secondary crushing, medium–low temperature drying, and screening, the mixture was separated into recycled coarse particles (>0.2 mm) and fine powder. This process successfully converted WRs into high-quality refractory raw materials with stable composition. Because this study was published before 2015, it is cited only as a historical example of early refractory recycling development and was not included in the formal systematic evidence synthesis, bibliometric analysis, or comparative assessment.

#### 4.1.2. Chromium-Containing Brick

The chromium-containing refractories are extensively applied in the metallurgical industry, cement industry and others because of their outstanding high-temperature properties. Their main failure forms include slag corrosion, thermal stress-induced spalling, and Cr^6+^ formation.

The recycling of spent chromium-containing refractory bricks mainly involves chromium stabilization, extraction and recovery of valuable components, and reuse of the latter as recycled aggregates or the raw material of new refractory production. The stabilization of chromium is the most important process that converts the toxic hexavalent form of this element to its less toxic or non-toxic forms. Studies have shown that the addition of stabilizers and control over the furnace atmosphere can be used to convert Cr (VI) to the more stable Cr (III) state and so solid-solve into the spinel phase, thus lowering its activity [39]. Peng et al. [40] have systematically reviewed chromium recovery technologies, and divided them into five large groups, including calcium roasting, sodium roasting, acid leaching, alkaline leaching, and sub-molten salt methods. Among these, conventional pyrometallurgical methods such as calcium roasting are uncomplicated and easily industrialized but produce highly hazardous CaCrO_4_-containing ash, which represents a serious environmental hazard.

Recent industrial work has demonstrated the feasibility of the closed-loop recycling of spent magnesia–chromite refractories. Previato Sardelli et al. [41] investigated the incorporation of recovered material from spent RH degasser linings into new direct-bonded magnesia–chromite bricks. The process involved removing the contaminated hot-face layer, selective cleaning, crushing, and quality control before the recovered material was incorporated into new refractory formulations. Recycled material contents of 25%, 50%, and 75% were evaluated, and the 25% formulation was subsequently validated in industrial production trials. This study demonstrates the practical potential of returning spent chromium-containing refractories to refractory manufacturing, while highlighting the importance of contamination control and consistent raw-material quality.

Despite this progress, the recycling of chromium-containing refractories remains constrained by the variability of spent materials, potential chromium-related environmental risks, and the need to maintain reliable high-temperature performance. Future work should therefore combine appropriate pretreatment and chromium-risk management with systematic evaluation of the physical, thermomechanical, and corrosion properties of recycled refractory products.

#### 4.1.3. Aluminum-Containing Brick

Aluminum-containing WRs are capable of being used in the manufacture of corundum bricks, high-alumina bricks, and other aluminum-containing shaped refractories.

Silva et al. [42] investigated the reuse of powders reclaimed from dismantled spinel-bonded high-alumina linings of steel induction furnaces in vibratable and self-flowing refractory castables. The recovered materials retained the microstructural characteristics associated with in situ spinel formation, while avoiding the disruptive expansion that can occur during the formation of spinel from separate raw materials. The mechanical performance after firing was consistently better for the self-flowing castables than for the vibratable systems. The study demonstrated the potential to incorporate 50–60 wt.% of cleaned refractory waste, indicating that appropriately processed high-alumina refractory waste can replace a substantial proportion of virgin refractory raw materials [42].

Similarly, Matinde and Msibi [43] investigated the use of reclaimed high-alumina refractory bricks recovered from the roof of an electric arc furnace in andalusite-based castables for tundish applications. The recovered refractory material was processed into graded aggregates and used to replace virgin andalusite at different proportions. The formulations containing up to 22 mass% reclaimed material exhibited flow behavior, open porosity, and bulk density comparable to those of the reference castable. However, high-temperature corrosion resistance and hot modulus of rupture indicated that the allowable replacement level depends on the required service conditions. The study demonstrated potential direct cost savings of up to 10% at a replacement ratio of 22 mass%, highlighting the importance of balancing recycled-material incorporation with refractory performance.

These studies demonstrate that the closed-loop recycling of aluminum-containing waste refractories can reduce the demand for virgin raw materials while maintaining suitable product properties. Nevertheless, the effectiveness of recycling depends on appropriate sorting, removal of contaminated layers, particle-size control, and the compatibility of the recovered material with the new refractory formulation. Further work should therefore focus on improving feedstock consistency and validating the long-term thermomechanical and corrosion performance of recycled refractory products.

#### 4.1.4. Silicon-Containing Brick

The biggest difficulty with the direct recycling of waste silica bricks is the stabilization of the high-temperature behavior of recycled silica aggregates to avoid a crack caused by residual stresses and phase transformations. As these recycled aggregates have a main crystalline form of tridymite and quartz, which can go through intricate phase transformations throughout their service, the reheating of these recycled aggregates could initiate uncontrolled volumetric change. Kenzhaliyev et al. [44] investigated microsilica-rich secondary raw materials for the preparation of mullite–silica refractories. Although the study did not use spent refractory bricks as the principal feedstock, it showed that amorphous SiO_2_ could promote secondary mullite formation and improve microstructural development. Therefore, this study is retained as contextual evidence concerning the use of silica-rich secondary raw materials, rather than as a core study of spent-refractory recycling. Frade et al. [45] investigated the recycling of industrial refractory brick waste to produce silica–alumina-based refractory bodies. By incorporating the waste into a mixture of clay, silica, and alumina, followed by wet processing and sintering at 1400 °C, the resulting specimens exhibited a modulus of rupture of approximately 63 MPa, demonstrating the feasibility of reusing refractory brick waste in the production of new shaped refractories.

#### 4.1.5. Magnesium-Containing Brick

Spent magnesia–alumina spinel bricks are known to be highly valuable recyclable refractories because of their excellent mechanical properties and structural stability at high temperatures. Wang et al. [46] employed a process analogous to the ‘coating’ technique in the pharmaceutical industry to recover hydrated magnesium aluminum spinel bricks. After calcination at 1100 °C, coating with alumina and sintering at 1600 °C, a magnesia–spinel composite material was obtained. This composite, combined with 97% high-purity sintered magnesia, gave a magnesia–alumina spinel brick with a bulk density of 2.85 g·cm^−3^ and a cold-pressed strength of 61.6 MPa. It had an alumina content of 4.85 wt.%, which was much less than that of the conventional bricks (11.87 wt.%). Both the recycled and conventional materials had a load refractoriness of over 1700 °C and withstood eight water-quenching thermal shock cycles, demonstrating highly comparable service behavior.

Magnesium–calcium bricks are widely used in cement kilns because of their excellent lining properties. However, their susceptibility to hydration limits their recycling. To address this, previous studies have proposed enhancing hydration stability through additive modification. Díaz-Tato et al. [47] significantly reduced the hydration weight gain rate from 3.07% to 0.01% by adding 1% iron spinel, while achieving a room-temperature compressive strength of 155 MPa for the recycled bricks. Xiong et al. [48] improved sintering density using 1.5% nano-Fe_2_O_3_, further increasing bulk density to 3.36 g·cm^−3^. These studies provide practical technical pathways to address insufficient hydration resistance during magnesia–calcium brick recycling.

Magnesium–iron–aluminum bricks possess both excellent kiln lining adhesion and thermal shock resistance. Research on recycling these spent refractories primarily focuses on restoring and further optimizing their service performance. Zheng et al. [49] demonstrated that, by using waste magnesium–iron–aluminum bricks as the main raw material, the kiln lining adhesion of recycled bricks can be significantly improved by rationally controlling the addition of electrofused iron–aluminum spinel (e.g., 10%). Furthermore, Kong et al. [50] successfully developed high-performance recycled magnesia–iron–alumina bricks through composite formulation design, achieving domestic substitution for this refractory material and demonstrating the engineering potential of recycling waste magnesia–iron–alumina refractories.

**Figure 8:**
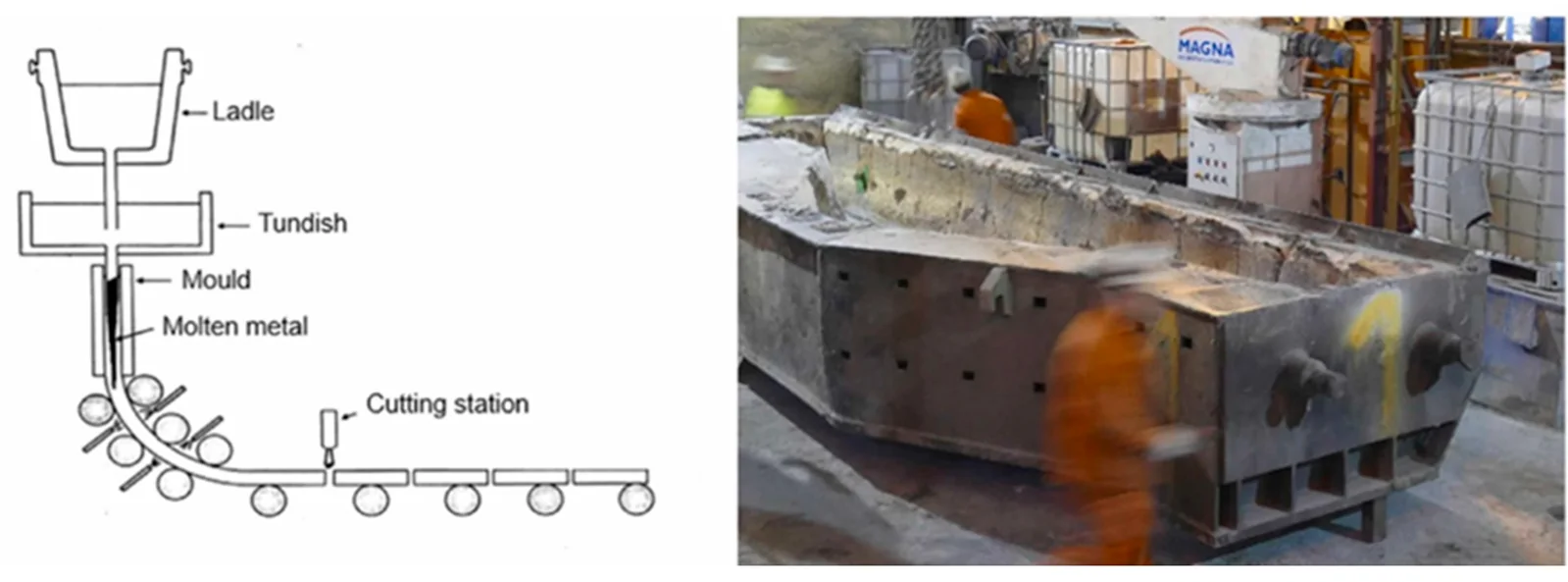
Schematic diagram of ladle involvement in the steelmaking process (**left**), morphology of MgO-containing refractory ladle (**right**) [51].

Beyond WR from the cement industry, the substantial volume of waste magnesia–carbon bricks generated by the steel industry also holds significant recycling value. As illustrated in Figure 8, ladles serve as critical vessels for transferring molten metal to molds during steelmaking. Their discarded refractories retain high magnesium oxide content.

Kusiorowski [52] investigated the technical pathway for converting waste magnesia–carbon bricks into high-performance MgO-ZrO_2_ refractory ceramics. By calcining waste bricks and blending them with zirconia before high-temperature sintering, the quality of secondary magnesia clinker was significantly enhanced. The resulting recycled material exhibits a dense structure with outstanding thermal shock resistance and erosion resistance. This research provides a novel approach for the high-value recovery of discarded magnesia–carbon bricks, demonstrating their cross-industry recycling potential to be transformed into high-quality basic refractories suitable for the glass or cement industries. Beyond waste magnesia bricks, waste materials from ladles—accounting for 42% of refractory consumption—represent another significant recyclable resource.

### 4.2. Regeneration of Unshaped Refractories

In the field of unshaped refractories, the preparation of traditional corundum raw materials consumes substantial resources, whereas recycled corundum produced from waste materials such as aluminum–chromium slag demonstrates significant substitution potential. Li et al. [53] compared the properties of recycled corundum with commercial electrofused corundum, finding that recycled corundum exhibits a lower thermal conductivity than white electrofused corundum, comparable sintering performance, and superior resistance to ladle slag corrosion. Thermodynamic calculations and experimental results indicate that CaO impurities present in the recycled raw materials promote liquid-phase sintering reactions above 1600 °C. After ladle slag corrosion, these impurities form larger amounts of CaAl_4_O_7_, which enrich the liquid phase environment and thereby increase the densification rate of material.

In addition to corundum-based materials, significant progress has been made in recycling silico-aluminous and magnesia-based waste. Schackow et al. [54] developed a novel refractory hydraulic binder using silico-aluminous waste from casting ladles. They found that this waste effectively replaced natural silica sand as a silicon dioxide source without altering the material’s thermal behavior. The major oxide contents of the control and waste-containing refractory mixtures are compared in Table 2 [54]. The incorporation of scrap improved particle grading; reduced voids between coarse particles, thereby enhancing bulk density and overall strength; and effectively suppressed slag and molten metal penetration. They found that replacing 25% by volume of commercial refractory patch blocks with silica and silica–alumina scrap could save over $50,000 annually.

Regarding magnesia waste, Dai et al. [55] utilized discarded magnesia refractory bricks from steel blast furnaces to prepare magnesium phosphate cement. The study revealed that the low reactivity of magnesium oxide in the waste significantly reduced hydration temperature rise and extended setting time, benefiting large-volume construction. When MgO content in the waste magnesia bricks reached 87%, the prepared cement exhibited a 28-day compressive strength approaching 60 MPa, along with excellent volume stability and long-term strength retention under water immersion conditions.

The selection of binders is critical for all refractory ceramics. For carbon-containing refractories, Stadtmüller et al. [56] explored green binder formulations utilizing lignin and collagen as alternatives to conventional phenolic resins. Thermogravimetric–mass spectrometry analysis revealed that this biomass binder system undergoes multistage thermal decomposition and oxidation between 250 °C and 640 °C. Although the recycled material exhibited a slightly higher porosity (13–19%) than conventional materials, this effect was effectively compensated by adding antioxidants such as Al and TiO_2_. Following the introduction of aluminum powder, in situ reactions at high temperatures generated carbides and nitrides.

4Al + 3C = Al_4_C_3_ (11)

However, it should be noted that the stability of aluminum carbide is limited to approximately 1400 °C. Above this temperature, it reacts with CO present in the atmosphere, resulting in the reduction of CO to carbon.

Al_4_C_3_ + 6CO = 2Al_2_O_3_ + 9C (12)

In the presence of nitrogen gas, aluminum carbide may also convert to aluminum nitride.

Al_4_C_3_ + 2N_2_ = 4AlN + 3C (13)

Moritz et al. [31] have utilized recycled magnesium oxide refractory bricks, which evidently still contain aluminum powder as an antioxidant, as evidenced by the presence of other phases such as Al_4_C_3_ and Al (OH)_3_. However, the presence of such phases should be evaluated in relation to the material composition, moisture exposure, and thermal history.

Stadtmüller et al. [56] identified five distinct steps in the oxidation process: (I) oxygen transport from the gas stream to the sample surface, (II) oxygen diffusion through the pore network, (III) oxygen reaction with graphite to form carbon monoxide, (IV) carbon monoxide diffusion from pores to the sample surface, and, finally (V) removal of carbon monoxide from the surface into the surrounding furnace atmosphere.

**Figure 9:**
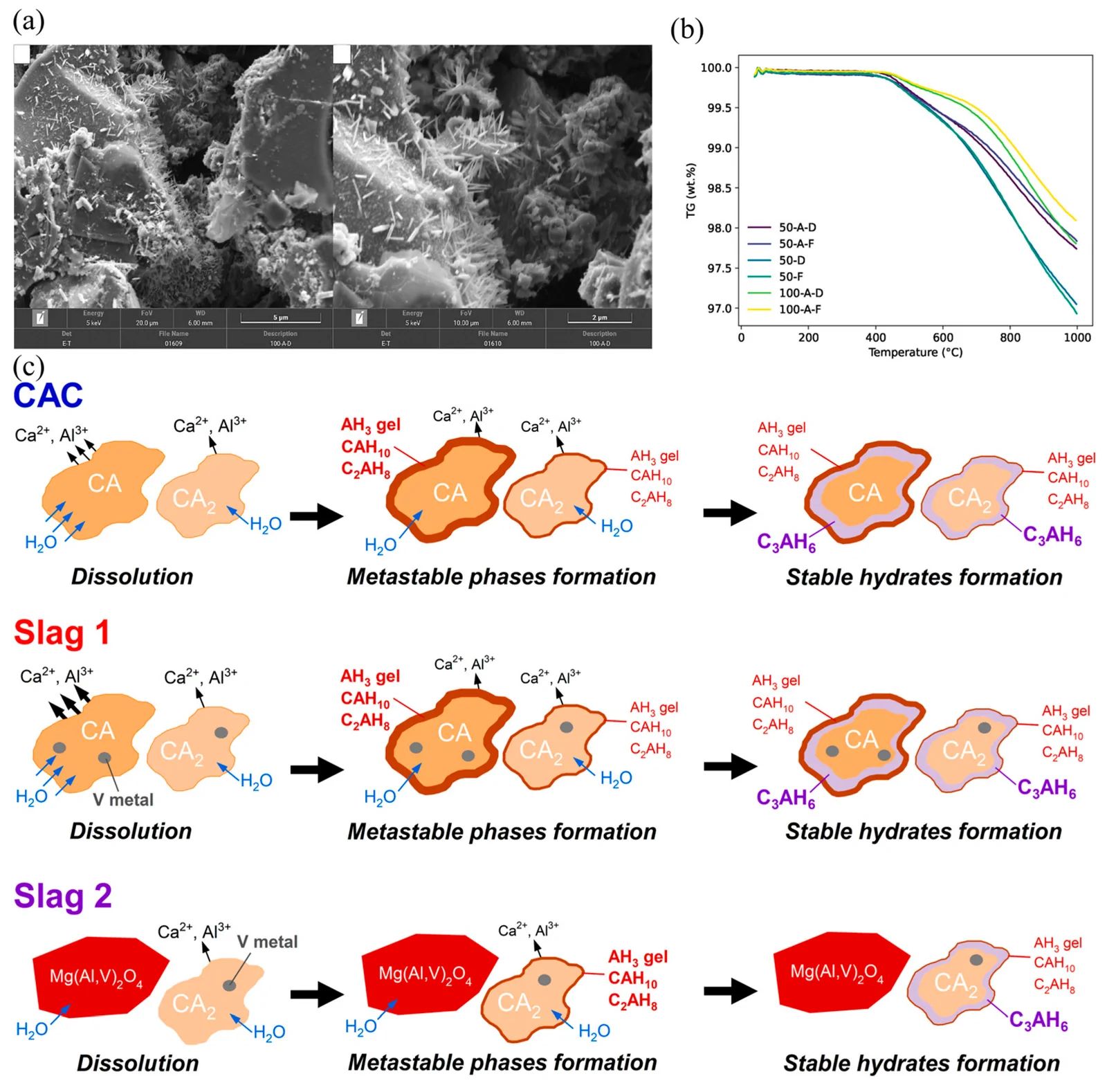
(**a**) SEM image of samples prepared from waste refractories and binder [56], (**b**) Oxidation behavior and post-test oxidation depths of different MgO–C formulations [56], (**c**) schematic illustration of the hydration pathways of typical CAC, Slag 1 and Slag 2 [58] (contextual slag–binder example; not included in the formal WR evidence base).

The binder system they proposed has been applied in ladles. Compared with the control group, batches containing waste MgO-C refractories typically exhibited higher open porosity, slightly lower bulk density, and reduced cold-pressed strength. However, the application of the antioxidant significantly enhanced the mechanical strength of the samples and provided effective oxidation resistance in an air atmosphere. SEM analysis revealed whiskers in the caked samples containing the antioxidant, as shown in Figure 9a [56]. As shown in Figure 9b, the oxidation-induced mass loss and post-test oxidation depths varied among the MgO–C formulations. The addition of Al and TiO_2_ improved oxidation resistance, indicating that appropriate additives can help maintain the service performance of refractories incorporating recycled materials [56].

Traditional magnesium–carbon refractories, despite their excellent performance, have long relied on hazardous asphalt or resin binders in their production, causing adverse environmental impacts. Addressing this issue, Lauermannová et al. [57] successfully developed a novel MgO-C refractory material by simultaneously employing a non-hazardous binder system and a high proportion of recycled spent MgO-C bricks. A mixture of lactose and tannin completely replaces conventional phenolic resins or coal tar pitch. Thermal analysis indicates that this binder system primarily releases water and carbon dioxide during heating, avoiding the issue of harmful substances like polycyclic aromatic hydrocarbons and formaldehyde produced by the pyrolysis of traditional binders. Furthermore, the crushed and screened spent magnesia–carbon bricks were graded into three size fractions (0–1, 1–3, 3–6 mm), substituting up to 50 wt.% of fresh magnesia aggregates. Samples with 50% recycled content exhibited slightly lower bulk density and cold crushing strength than reference samples prepared entirely from virgin raw materials. The key to achieving the hydration process is to control the reaction of calcium aluminate, magnesium oxide, and other substances with water to form metastable or steady-state hydrates (Figure 9c) [58].

## 5. Open-Loop Recycling

Open-loop recycling refers to the conversion of WRs into non-refractory materials with high utilization value via physical, chemical or metallurgical processes, which is different from closed-loop recycling that retains the refractory properties of WRs.

### 5.1. Recycling into Metallurgical and Chemical Materials

Recycling WRs into metallurgical and chemical materials is the most mature open-loop recycling direction, and it mainly utilizes the main chemical components (MgO, CaO, Al_2_O_3_, etc.) and valuable metal elements in WR. This section is divided into two parts: flux agent and metal recovery.

#### 5.1.1. Flux Agent

Utilizing WRs as flux agents fundamentally involves macro-level utilization of their primary chemical constituents. This approach features relatively low technical barriers and serves as a rapid pathway for achieving on-site recycling. Basic bricks like dolomite and magnesia are commonly used as slag formers or modifiers in metallurgical processes.

In traditional metallurgical processes, lime and dolomitic lime are added to electric arc furnaces to increase MgO saturation in the slag, thereby reducing refractory erosion. Replacing dolomite and lime with waste MgO-C refractories has been demonstrated to increase MgO content in slag and extend refractory life, provided appropriate dosages are carefully determined. Wang et al. [59] obtained similar results through industrial trials at a South Korean steel company, and confirmed that waste MgO-C refractories can successfully substitute natural dolomite as a flux agent during the steelmaking process.

Understanding the dissolution behavior of waste MgO-C bricks in molten slag is essential for their reuse as slag-conditioning materials. Liang et al. [60] discovered that the dissolution behavior is influenced by FeO content and temperature. With higher levels of FeO in the slag, graphite carbon in the refractory material is effectively oxidized. This process disrupts the original dense structure of the refractory, significantly accelerating the dissolution of MgO into the slag phase. Meanwhile, high temperatures supply extra energy to the dissolution process, increasing the total dissolution rate of MgO-C refractories.

Konovalova et al. [61] developed a process for preparing converter smelting flux from WRs. This method involves mixing crushed WRs with particle sizes under 10 mm with magnesia filler and silicate binder, then forming them into compacted flux blocks via a semi-dry vibration compaction process. The prepared flux has an apparent density of 2.2–2.5 g·cm^−3^ and sufficient mechanical strength to meet transportation and handling requirements. Avelar et al. [62] investigated the recycling of spent MgO-C refractory bricks removed from electric arc furnaces as slag-conditioning materials. The crushed refractory waste was evaluated alongside dead-burned dolomite fines, with particular emphasis on slag foaming behavior. Their results demonstrated the potential of MgO-rich refractory waste for slag conditioning in steelmaking, providing an alternative recycling pathway for spent basic refractories.

#### 5.1.2. Metal Recovery

Spent magnesia–chrome refractories often contain valuable elements, including chromium and magnesium, as well as metals introduced during smelting, such as lead, silver, and bismuth. Importantly, hydrometallurgy can be used to selectively leach target metals. Jiao et al. [63] developed a method based on acid leaching, alkali precipitation, and evaporation to recover valuable chromium and magnesium from waste magnesia–chromite brick. The optimal reaction conditions were observed at a hydrochloric acid concentration of 5.0 mol/L, liquid-to-solid ratio of 5:1, leaching temperature of 343 K, leaching time of 90 min, and stirring speed of 400 rpm. Under these conditions, 95.14% of the Cr was retained in the slag phase as chromite, which had the properties required for direct smelting. Subsequently, adjusting the leachate pH to 7.0 using Mg (OH)_2_ removed over 90% of impurities, including Cu, Pb, and Bi. High-purity magnesium chloride hexahydrate was then obtained from the final solution via evaporation. Xue et al. [64] further employed a HCl-FeCl_3_ composite system. By operating at 4.0 mol/L HCl, a liquid-to-solid ratio of 7:1, 4% FeCl_3_, and 5000 rpm for 90 min at 70 °C, they achieved Cr, Fe, and Al enrichment in the residue (recovery > 92%), while valuable metals like Mg, Pb, Bi, Ag, and Sb were efficiently enriched in the leachate (all leaching rates exceeding 89%), validating a highly efficient approach for multi-metal separation.

For metals present in physical form within waste materials, physical beneficiation methods prove more economical. Xue et al. [65] achieved comprehensive utilization of spent MgO-Cr_2_O_3_ refractories through gravity separation and flotation, successfully recovering 99% of silver, 82% of lead, and 81% of bismuth from the WR. The obtained concentrate can be directly fed back into the smelting system as feedstock, while the low-impurity tailings can serve as raw material for new refractory products. For MgO-Cr_2_O_3_ refractories discarded from converter operations in copper smelters, Han et al. [66] developed an efficient flotation process to recover enriched metallic copper. The phase analysis showed that Cu is present as Mg_1-x_CuO, Cu_2_O, and metallic Cu as part of a periclase–chromite matrix. The hydrophilic nature of the oxide matrix causes copper oxides to enter the pores of brick, making recovery difficult. Through systematic optimization of the flotation conditions (grinding time, emulsified kerosene, sodium isopentyl xanthate, and Na_2_S), they achieved a copper recovery rate of 95% and a concentrate grade of 21.4%.

Pyrometallurgy is applicable for removing impurities or transforming materials by thermal treatment. Xue et al. [67] employed chlorination volatilization to selectively volatilize impurity metals (Pb, Bi, Cu, etc.) in mineral processing tailings in the presence of N_2_ gas to obtain a purified oxide slag containing magnesite and chromite which will be recovered later. After systematically evaluating the effects of calcination temperature, time, atmosphere, and MgCl_2_ dosage, they found that elevated temperatures markedly enhanced the volatilization efficiency under a N_2_ atmosphere. At 1100 °C with 9% MgCl_2_ for 40 min, the process suppressed the total impurity content to under 1%, and metal volatilization rates were as high as 99%. This deep purification was also confirmed by an 800 g scale-up test.

Moreover, beyond extracting valuable metals from Cr-Mg refractories, WRs with high alumina content also present significant recycling potential. Hou et al. [68] applied WRs from aluminum electrolytic cell overhauls as raw material to produce Al-Si alloys directly through electrolytic precipitation. Pilot studies reveal that mixing scrap with alumina at a 1:9 ratio enables stable operation in 400 kA large-scale electrolytic cells. Although this process slightly elevates energy consumption, it delivers substantial resource dividends by reducing fresh alumina consumption by 100 kg/t of aluminum.

### 5.2. Recycling into Building Materials

The application of WRs in the building materials sector represents a key pathway for achieving their large-scale resource recovery. Based on the role these materials play within the building materials system, they can be primarily categorized into three categories: cement, aggregates, and ceramics.

#### 5.2.1. Cement

Recycling WRs into cement represents a sustainable approach to resource utilization. MgO-rich WRs can be incorporated into magnesium phosphate cement (MPC) as a substitute for dead-burned magnesia. Wang et al. [69] prepared MPC using recycled magnesia as a full substitute for dead-burned magnesia. Due to its higher crystallinity and CaMgSiO_4_ impurities, the recycled MgO (49.56 μm) exhibited lower hydration activity than dead-burned magnesia (37.35 μm). However, at a Mg/P molar ratio of 4, the recycled MPC achieved high compressive strengths of >65 MPa (28 d). The low-reactivity unreacted particles functioned as skeletal fillers, significantly reducing long-term drying shrinkage and enhancing volumetric stability. Despite a slightly lower 120 d water-resistance strength retention of 67.9%, the recycled MPC demonstrated excellent engineering viability.

**Figure 10:**
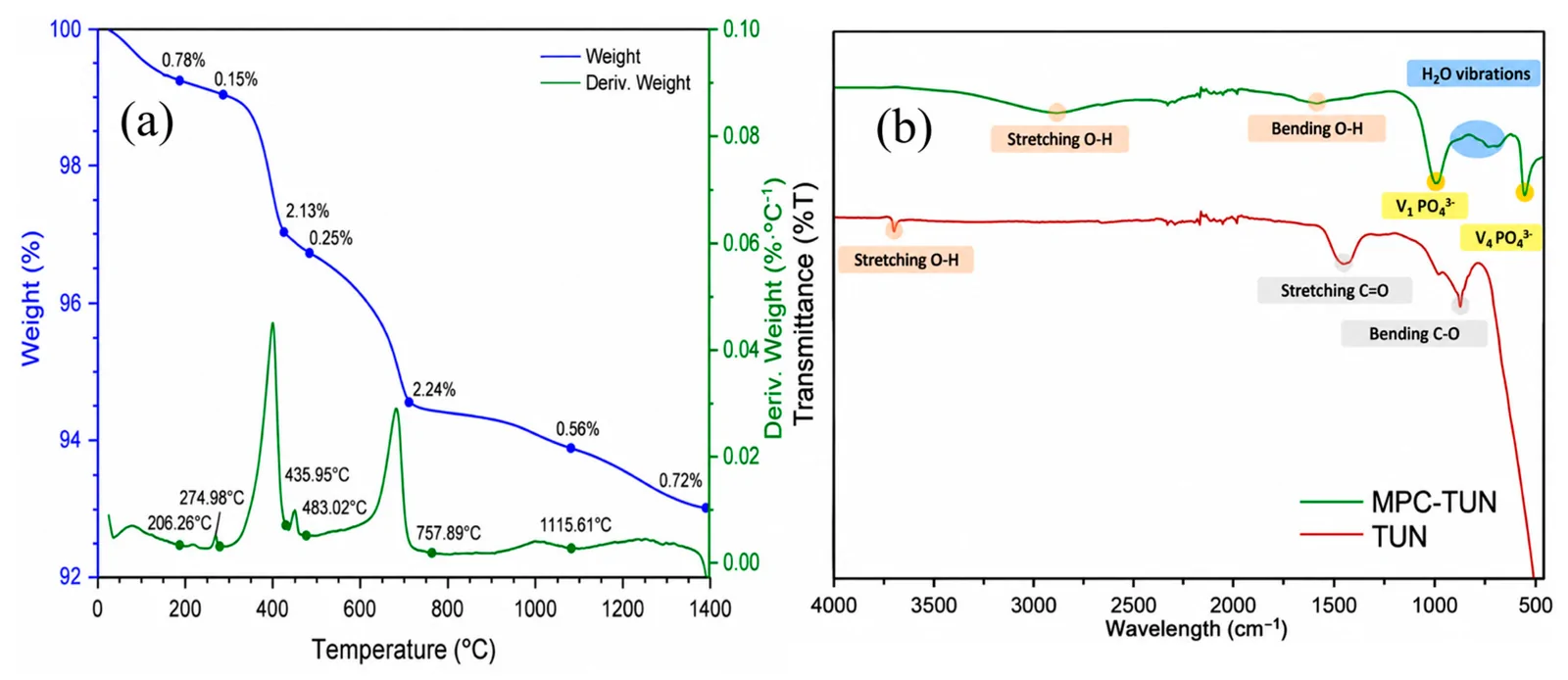
(**a**) Thermogravimetric analysis of waste ladle refractories [51] and (**b**) infrared spectra of scrap refractories from ladles and prepared magnesium phosphate cement [51].

Alfocea-Roig et al. [51] evaluated the feasibility of preparing MPC using WR from a steel plant’s ladle (MgO content: 68.70%). The best formulation was found to be 60% waste, 40% KH_2_PO_4_, and 1% H_3_BO_3_, which gave a 28 d compressive strength of about 27 MPa and a flexural strength of about 7 MPa. The reaction mechanism was also determined by FTIR analysis (Figure 10b), which showed changes in the Mg-O bond vibration and the emergence of characteristic phosphate (PO_4_^3-^) and crystalline water peaks at 1000–1200 cm^−1^. The primary hydration product was MgKPO_4_·6H_2_O. Nevertheless, leaching experiments showed that levels of Se were higher than EU non-hazardous waste levels, requiring the use of hazardous waste treatment at end-of-life. This highlights the importance of regulating toxic heavy metal leaching in future applications.

El-Didamony et al. [70] studied the pozzolanic behavior of WR powder combined with CaO and cement. Measurements of chemically bound water content and free lime consumption indicated that reactive silico-aluminous components in WR reacted with calcium hydroxide (CH). Cement substitution experiments (5–20 wt%) showed that, after 90 days, pastes containing 5–10% WR had compressive strengths higher than those of pure cement because of microstructure refinement. Conversely, a 20% substitution induced a slight strength decline via clinker dilution. These findings validate the potential of silico-aluminous WRs as sustainable building materials.

With its outstanding high-temperature resistance, corrosion resistance, and rapid hardening properties, recycled cement from WRs demonstrates broad application prospects in construction and industrial sectors. In high-temperature industrial environments, it can be used for lining blast furnaces in steel mills and for the construction and repair of industrial kilns. In infrastructure development, its high strength and durability make it suitable for high-load roads, bridge structures, and anti-corrosion projects in seaports and docks. Furthermore, this material is suitable for manufacturing heat-resistant and corrosion-resistant concrete in specialized environments such as chemical plants and power stations. The other application is in the construction of power plant chimney lining and waste incineration plant refractory structures, with the ability to combat both temperature extremes and chemical corrosion problems. Recycled cement based on WRs as a green building material that is compatible with circular economy concepts not only allows one to make emergency repairs of the aging industrial facilities quickly but also allows for the manufacture of prefabricated elements of green buildings.

However, this approach imposes stringent requirements on the chemical composition and fineness of the waste material, resulting in relatively high processing costs.

#### 5.2.2. Aggregate

In contrast, a simpler and more scalable utilization method involves upcycling this waste into recycled aggregate.

The application of WRs as recycled aggregates requires careful evaluation of chemical stability and volume stability. Potential issues, such as heavy metal leaching, delayed hydration expansion of MgO-rich materials, and hydration instability of dolomite refractories, may limit their direct utilization. Despite these challenges, WRs like magnesia, MgO-C, magnesia–alumina, and zirconia-based materials have been extensively studied in terms of substituting natural aggregates in high-performance mortars and concrete due to their excellent physical and mechanical properties. Wang et al. used crushed magnesia bricks (MgO content > 82%) as raw material to prepare MPC mortar. They achieved a 28-day compressive strength of 68.5 MPa. The recycled MgO particles contributed to strength development and volume stability due to their relatively low hydration reactivity.

In concrete and mortar preparation, WR aggregates often impart unique mechanical properties to the cementitious matrix. Aboutaleb et al. [71] investigated the potential of using recycled zirconia bricks (containing approximately 40% zirconia and 12% SiO_2_) as fine aggregates in self-compacting mortar (SCM) to partially or fully replace natural sand. The self-compacting mortar achieved compressive and flexural strengths of 35 MPa and 7 MPa, respectively, indicating the potential of zirconia-based WRs as a stable aggregate source for cementitious materials. The physical properties of natural sand and recycled refractory brick aggregates are compared in Table 3. The recycled aggregates exhibited higher water absorption and specific surface area, which may influence the workability of cementitious mixtures [71]. Similar results have been reported by Ghosh et al. [72], where recycled WR aggregates improved early-age compressive strength, with optimal replacement ratios of 10–20%.

Malenšek et al. [73] further prepared alumina-rich WR aggregates through cold bonding, demonstrating that processed aggregates could achieve improved mechanical performance after firing.

For the MgO-C refractory bricks in industrial production, Arianpour et al. [74] proposed a pretreatment process to remove the surface slag erosion layer and decarburize at 1400 °C. Although the recycled aggregates exhibited microcracks and reduced density, the addition of 30 wt.% recycled aggregate maintained acceptable mechanical and physical properties without adverse effects. However, residual impurities such as CaO and SiO_2_ at grain boundaries reduced slag corrosion resistance, indicating the importance of impurity removal and pretreatment before reuse.

The high-temperature stability of WR aggregates provides advantages for their application in heat-resistant concrete. Ordinary concrete develops cracks at temperatures exceeding 300 °C due to the dehydration of the C-S-H gel and thermal expansion of aggregates, resulting in strength degradation. However, Ghosh et al. [75] noted that concrete containing recycled refractory aggregates showed much improved compressive strength after exposure to temperatures of 200–600 °C, where there was a 67% improvement at 400 °C. In the higher temperature range of 800 °C to 1000 °C, samples with high replacement rates of 70–100% had better mechanical retention. Other than fine aggregates, waste refractory bricks can be used as coarse aggregates. Similarly, Khattab et al. [76] reported that WR brick aggregates reduced thermal cracking by providing additional pore channels for steam release. They also reported improved elastic modulus retention after high-temperature exposure [77].

Sinha et al. [78] employed Portland slag cement as the binder material and used crushed fine waste refractory bricks as the substitute for natural fine aggregates to prepare sustainable concrete samples. River sand was used as natural fine aggregate and crushed granite as natural coarse aggregate in comparative experiments. They found the best coarse aggregate gradation ratio was 60% 12.5–20 mm and 40% 6.25–12.5 mm of coarse aggregate. The cement: fine aggregate: coarse aggregate proportion was fixed at 1:1.98:3.78 and mixing was done at a steady water–cement ratio of 0.45. The mixed paste was poured into molds for cubes, cylinders, and prisms. After full immersion in water and curing for 7 days and 28 days, the specimens underwent compression, splitting tensile, and flexural tests.

In the above-described study, the control group achieved a compressive strength of 36.13 MPa at 28 days. Samples incorporating waste refractory bricks as fine aggregate showed increased compressive strength at 7 days. However, after the 28-day curing period, this trend did not persist. Only samples with the highest waste refractory brick content (30%) exhibited a linear increase in compressive strength. Compared with the control group, the compressive strength gains for samples incorporating waste refractories as fine aggregate at 10 wt.%, 20 wt.%, and 30 wt.% were 6%, 8%, and 3%, respectively. The splitting tensile strength of the control concrete cylindrical specimens after 28 days of curing was 3.07 MPa. The splitting tensile strength of concrete incorporating waste refractories as fine aggregate increased by 7%, 7%, 8%, and 1% at 10 wt.%, 20 wt.%, and 30 wt.% additions, respectively. Compared with ordinary concrete specimens, the self-weight of the specimens slightly increased due to the replacement of natural river sand with recycled aggregate from discarded refractory bricks.

Nematzadeh et al. [79] investigated the high-temperature performance of concrete incorporating recycled refractory aggregates prepared with Portland cement and calcium aluminate cement. The results showed that, although mechanical properties decreased with increasing temperature, recycled refractory aggregates contributed to improved thermal stability compared with conventional concrete. The same group further evaluated the durability of recycled refractory aggregate concrete under acidic conditions [80]. The results indicated that calcium aluminate cement and PVA fiber reinforcement improved acid resistance, whereas the use of recycled refractory fine aggregates alone showed limited corrosion resistance. After 63 days of sulfuric acid exposure, the optimized specimens still maintained a compressive strength of 38.6 MPa, with a mass loss of 1–5%, highlighting the need for the application-specific evaluation of chemical durability.

**Figure 11:**
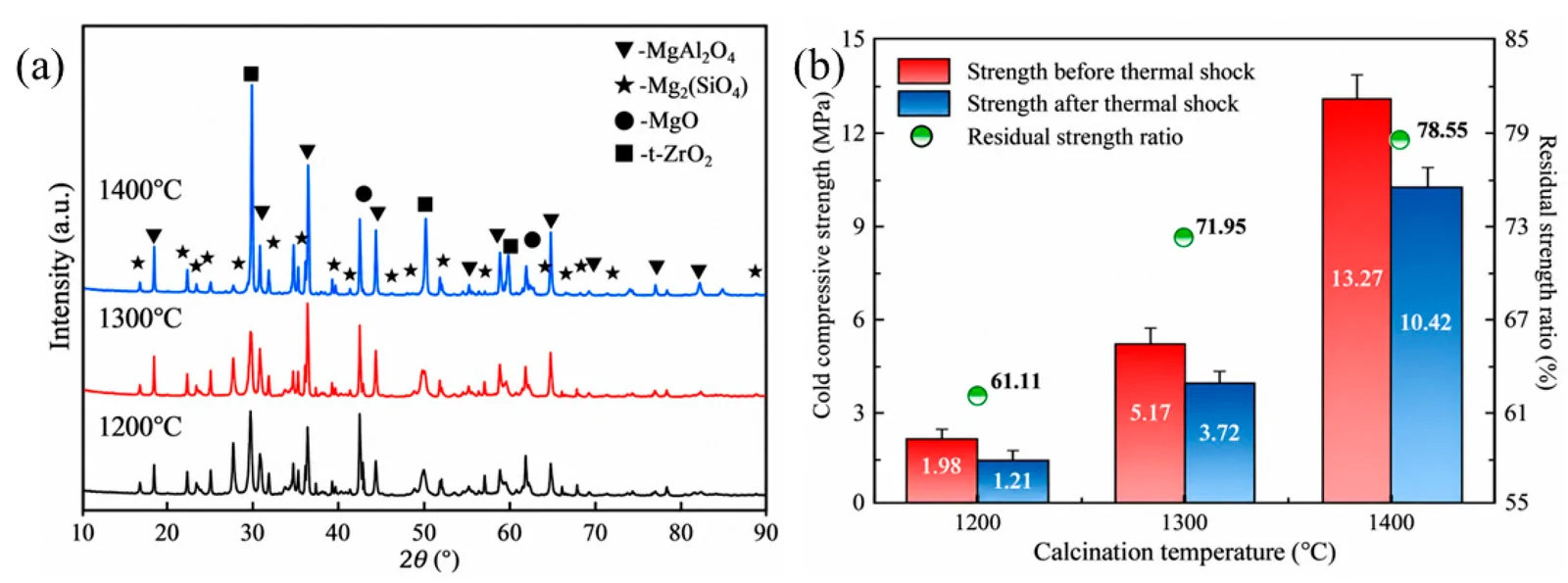
(**a**) XRD patterns of the sample with different sintering temperatures [81] and (**b**) compressive strength and thermal shock resistance of samples [81].

In an attempt to improve the properties of recycled aggregates, researchers explored techniques for preparing lightweight, high-strength aggregates through sintering modification. Al_2_O_3_-ZrO_2_-C refractories incorporated with light-burned magnesium oxide can develop aggregates at elevated temperatures [81]. With the calcination temperature increased from 1200 °C to 1400 °C, the intensity of the MgAl_2_O_4_ diffraction peak became more intense (Figure 11a), which indicates that there is a solid-phase reaction between alumina and magnesium oxide. As shown in Figure 11b, the aggregate developed at 1400 °C had the highest compressive strength (13.27 MPa). Upon being subjected to three thermal shock quenches, it attained a residual strength ratio of 78.55%. This performance is attributed to the abundant formation of the MgAl_2_O_4_ phase, which possesses a low thermal expansion coefficient and effectively mitigates thermal stresses.

In addition to conventional uses of thermal engineering, WR aggregates offer opportunities in functional pavements and green materials. Xue et al. [82] used the high hardness and abrasion resistance of high-alumina refractory waste to apply high-friction surface treatment, which confirmed that they can be used to give good long-term anti-skid texture. Seco et al. [83] prepared aggregates based on ladle and converter scrap in carbon sequestration, finding that CO_2_ curing is capable of promoting carbonation reactions, decreasing water absorption and increasing the strength of the particle. At present, the recycled aggregates from WR are widely applied in steel metallurgy, cement and glass industries, non-ferrous metal smelting, and construction, emerging as a major route for resource utilization of high-temperature industrial solid waste.

In summary, recycled cement and recycled aggregates are both parts of the resource utilization system of WRs in the building materials industry. Cement production primarily exploits the chemical reactivity of WRs, whereas aggregate production utilizes their physical properties and capacity to form a load-bearing skeleton. These complementary approaches accommodate WRs with different compositions and characteristics. These methods complement each other and offer appropriate solutions to WRs of different qualities and types.

#### 5.2.3. Ceramic

Through solid-phase reactions at high temperature sintering, some WRs are converted into ceramics with superior mechanical, thermal, or chemical properties. Ceramic concrete is another term used to describe chemically bonded ceramics. Conventional chemically bonded ceramics are formed via acid-base neutralization reactions between MgO and KH_2_PO_4_, giving rise to a primary crystalline phase having a magnesium potassium phosphate (MgKPO_4_·6H_2_O) structure. Recently, there has been an increasing interest in chemically bonded ceramics based on industrial waste. Luza et al. [84] synthesized porous chemically bonded ceramics using waste MgO-C refractory bricks from electric arc furnaces. The resulting MgKPO_4_·6H_2_O-based ceramics exhibited compressive strengths of 1.0–1.4 MPa and high porosity (60–74%), demonstrating the feasibility of converting WRs into functional ceramic materials. Kusiorowski [52] has observed that despite the widespread use of magnesia refractories and the substantial quantities of waste generated in the steel, glass, and cement industries, their recycling remains largely limited to use as coarse aggregates (with addition rates typically not exceeding 30%). The utilization rate of fine aggregates remains extremely low due to the difficulty in separating impurities, resulting in waste of critical non-renewable magnesite resources. Therefore, this study proposes a high-value utilization pathway for preparing zirconia-modified magnesia refractories using discarded MgO-C bricks.

The study utilized waste MgO-C bricks from ladles, which were pretreated to remove the contaminated layer and recover secondary magnesia materials. This was then combined with monoclinic zirconia and commercial magnesia, sintered at 1660 °C to prepare MgO-ZrO_2_ composite ceramics. The resulting MgO-ZrO_2_ ceramics exhibited improved corrosion resistance against glass melts. This was attributed to the low porosity and the excellent uniformity and dispersion of zirconia compounds within the molten material. Furthermore, the addition of zirconia enhanced the cold-pressed strength of the ceramics, particularly in the case of the fused MgO-ZrO_2_ co-clinker, while also improving corrosion resistance. These results demonstrate the potential of discarded magnesia refractories as secondary raw materials for producing high-value ceramic products.

Recycled ceramics made from WRs have potential applications in high-temperature and corrosion-resistant environments due to their inherent thermal stability and chemical resistance. However, further studies are required to evaluate their long-term performance, scalability, and economic feasibility before large-scale industrial implementation.

### 5.3. Recycling into Functional Materials

WRs have potential in functional materials because of their specific chemical compositions. Firstly, using their chemical purity, they may be reused as a pollutant degrader in an organic form. Secondly, taking advantage of their large heat capacity and phase change behavior, they can be reused as thermal storage materials.

#### 5.3.1. Organic Pollutant Degrading Agent

To treat phosphate- and ammonia-nitrogen-containing industrial wastewater, Li et al. [85] used WRs as a magnesium source. Acid leaching yielded Mg^2+^-rich solutions used to induce magnesium ammonium phosphate precipitation. At the optimal pH of 9.5, the removal rates of phosphate and ammonia nitrogen reached 99.6% and 98.2%, respectively. Preliminary economic assessments indicate considerable cost savings compared with the use of high-purity chemical reagents. These results demonstrate the potential of WR for combined solid waste and wastewater treatment and resource recovery.

Building on this foundation, functional materials derived from WR have been further expanded in water treatment applications. Kuzin et al. [86] prepared a composite magnesium–titanium coagulant by dissolving WRs in a titanium sulfate system. This coagulant demonstrated excellent removal capabilities for ammonium ions, phosphates, and dissolved organic matter in plastic reprocessing wastewater, outperforming traditional iron-based or aluminum-based coagulants in overall performance. Uysal et al. [87] utilized WR powder as a reinforcing skeleton, combined with biopolymers to prepare hydrogel adsorbent materials, achieving over 98% removal efficiency for Cu^2+^ in wastewater. Furthermore, Hammal [88] investigated the incorporation of spent chamotte refractory materials recovered from cement kiln linings into geopolymer membranes for pharmaceutical wastewater treatment. By replacing 20% of calcined clay with chamotte waste, the prepared membrane achieved a COD removal efficiency of 95.74% through a two-stage process combining adsorption and membrane filtration. These findings demonstrate the potential of spent refractory materials as alternative raw materials for wastewater treatment applications.

It is noteworthy that not all solid wastes containing organic pollutants are suitable for treatment through degradation or removal methods.

#### 5.3.2. Thermal Storage Material

Beyond structural ceramic applications, the thermal properties of some WR materials also enable their utilization as thermal storage media.

The scarcity of clean drinking water has spurred the development of solar seawater desalination technologies. Introducing thermal storage materials to release stored heat at night has become a key strategy for enhancing efficiency. Panchal et al. [89] explored the potential of waste magnesia refractory bricks as a heat storage medium. This material has high density (3 g/cm^3^), good thermal conductivity (5.0 W/m·K), high specific heat capacity (1130 J/kg·K), and excellent corrosion resistance. Comparative experiments were conducted with single-slope solar distillers containing 0, 3, and 6 kg of waste magnesia refractory bricks, and it was observed that the addition of waste magnesia bricks changed the thermodynamic behavior of the system and that the peak thermal efficiency was attained after 1 h.

The application of recycled refractories as thermal storage materials is still at an early stage. Their high-temperature stability and thermal properties indicate potential for energy-related applications; however, further studies are required to evaluate their long-term thermal cycling performance, scalability, and economic feasibility.

### 5.4. Comparative Assessment of Waste Refractory Recycling Technologies

To systematically compare the recycling technologies discussed above, representative studies were selected based on WR types, recycling pathways, and the availability of reported performance data. As summarized in Table 4, these studies were compared in terms of waste type and origin, pretreatment methods, recycling pathways and products, recycled fraction or recovery efficiency, key performance indicators, validation scale, environmental and economic evidence, and main limitations. Environmental and economic performance was assessed based on the available quantitative evidence reported in the original studies.

## 6. Conclusions, Challenges, and Limitations

This review systematically analyzes the physical and chemical failure mechanisms of WR materials and clarifies their closed-loop and open-loop recycling pathways. The research results indicate that the applicability of the recycling methods is not only dependent on the original composition of the refractory materials, but also closely related to the structural and chemical changes that occur during their use. Thermal shock and mechanical damage usually lead to cracking, spalling, and fragmentation, whereas slag penetration, oxidation, and interfacial reactions may alter phase composition and introduce impurities. These changes directly affect the subsequent pretreatment requirements and the feasibility of material regeneration.

The relationship between the failure mechanism and the recycling strategy provides an important basis for selecting the appropriate recycling path. Refractory waste materials with relatively stable chemical composition and low pollution are more suitable for closed-loop recycling through processes such as sorting, crushing, size classification, and binder modification. In contrast, materials after chemical failure need to further remove impurities or stabilize the phase state before reuse. When the requirements for material performance and environmental safety cannot be satisfied for closed-loop recycling, open-loop recycling technologies can provide alternative applications in metallurgical processes, building materials, and functional products. Therefore, when choosing the recycling path, the state of the recovered materials and their expected application should be considered simultaneously.

Although recycling technology has made significant progress, there is still a gap between laboratory feasibility and industrial implementation. Most studies remain limited to laboratory experiments, while pilot-scale demonstrations and industrial applications are still insufficiently presented. Moreover, although some studies have mentioned material conservation and potential economic or environmental benefits, quantitative life cycle assessment and techno-economic evaluation are still limited.

The main challenges faced by large-scale recycling include the difficulty in separating impurities from refractory waste materials, inconsistent quality of recycled products, and the lack of standardized evaluation criteria. In addition to technological progress and process optimization, systematic classification and management of WRs is also a key prerequisite for achieving efficient resource utilization. Establishing unified classification standards and identification systems helps accurately characterize waste materials, distinguish their inherent characteristics and potential utilization value, and provide guidance for selecting the appropriate recycling path. At the same time, industrial management and regulatory frameworks should be improved to promote standardized recycling practices and prevent resource waste and secondary environmental pollution. Through the integration of precise classification, targeted regeneration strategies, standardized evaluation systems, and industrial-scale verification, WR recycling is expected to shift from low-value waste treatment to multi-level resource utilization, thereby reducing reliance on primary raw materials, reducing environmental burdens, and promoting the circular transformation of the high-temperature industry.

## Figures and Tables

**Table 1:** Average chemical composition of refractories before and after use (%) [5].

Types of Refractory Materials	Service Status	MgO	CaO	Fe_2_O_3_	Al_2_O_3_	SiO_2_	C
MgO-C	U	96.2	1.3	0.7	0.8	0.9	11.0
	S	94.5	1.8	0.9	1.1	1.3	10.0
MgO-C (electric arc furnace)	U	84.7	<1.2	<0.5	<0.5	<0.5	12.7
	S	86.1	3.6	4.7	0.7	1.7	15.3
MgO-C (steel ladle)	U	90.5	<1.2	<0.5	<0.5	<0.5	9.3
	S	79.4	6.1	1.8	5.1	3.8	9.3
Fired MgO	U	97.0	0.8	0.4	1.3	0.4	0.1
	S	90.0	2.0	1.5	1.5	3.5	/
Fired doloma	U	39.0	59.0	1.0	0.5	1.0	/
	S	35.0	55.0	3.0	3.0	4.0	/
Doloma–C	U	39.0	58.0	0.6	0.6	0.9	5.3
	S	37.0	59.0	0.7	1.4	1.3	4.6
Fired andalusite	U	0.3	<0.15	0.9	59.0	38.0	/
	S	0.7	0.4	1.6	58.0	37.0	/
Fired bauxite	U	0.5	0.5	1.4	83.0	10.0	/
	S	0.7	1.0	1.8	79.0	14.0	/
Fired chamotte	U	0.5	<0.15	1.3	43.0	53.0	/
	S	1.5	/	2.0	35.0	54.0	/

Note: U represents unused, S represents spent. Data in the table are derived from the literature. The specific composition of refractories may vary depending on type and application.

**Table 2:** Major oxide contents of the control and waste-containing refractory mixtures determined by XRF (wt.%) [54].

Mixture	SiO_2_	Al_2_O_3_	Fe_2_O_3_	CaO
MP (control)	27.99	62.00	1.45	6.31
RS/RSA-1, 25%	29.03	57.49	1.03	8.64
RS/RSA-1, 75%	35.49	52.93	1.35	8.56
RS/RSA-1, 100%	36.57	51.24	1.43	8.78
RS/RSA-2, 25%	24.24	59.86	1.03	7.65
RS/RSA-2, 75%	24.02	64.34	1.64	7.03

**Table 3:** Physical properties of natural sand and recycled refractory brick aggregates [71].

Property	Natural Sand (NS)	Refractory Brick Wastes (RBW)
Apparent density (kg/m^3^)	1520	1180
Specific gravity (kg/m^3^)	2500	2670
Water absorption (%)	1.03	3.01
Specific surface (m^2^/kg)	6.24	6.67

**Table 4:** Comparative assessment of representative waste refractory recycling technologies.

Reference	Feedstock and Pretreatment	Recycling Pathway and Product	Performance and Validation	Environmental and Economic Impact; Limitations
[58]	Waste MgO-C bricks; crushed and graded.	Recycled MgO-C refractories (closed-loop regeneration).	Up to 50 wt.% virgin feedstock replaced; laboratory characterization of physical and mechanical properties.	Not mentioned. The long-term service performance has not yet been verified.
[45]	Silico-aluminous brick waste; wet mixing, pressing and firing at 1400 °C.	Silico-aluminous refractory bricks (closed-loop regeneration).	Flexural strength ≈ 63 MPa and linear shrinkage ≈ 6%; laboratory specimens.	Not mentioned. The service life has not been evaluated.
[55]	Waste foundry linings; removal of adhering contaminants, crushing and grading.	Hydraulic-setting refractory patch material (closed-loop regeneration).	Silica sand replaced at 25, 75 and 100 wt.% in laboratory formulations.	Estimated savings of US$55,653 per year in one foundry scenario; economics depend on site conditions.
[63]	Waste MgO-C bricks; crushed and blended with dolomite fines, then briquetted.	Conditioner for electric arc furnace slag (open-loop recycling).	Waste bricks comprised 23–45 wt.% of tested briquettes; maximum slag foam height 5.5 cm in laboratory tests.	Not mentioned. The industrial verification situation was not explained.
[69]	Waste aluminum-cell refractories; separated, crushed and milled.	Al-Si master alloys produced by electrolysis (open-loop recycling).	Approximately 100 kg less alumina consumed per ton of metal. Pilot-scale test.	Reduced alumina demand was accompanied by higher electricity consumption. No quantitative LCA was mentioned.
[71]	Waste aluminosilicate refractory bricks from a cement plant; crushed and finely ground.	Blended Portland cement with recycled brick powder (open-loop recycling).	At 5–10 wt.% cement replacement, compressive strength exceeded that of plain cement up to 90 days; laboratory tests.	Not mentioned. Industrial verification was not mentioned.
[86]	Waste steelmaking refractory bricks; acid leaching and chemical separation.	Phosphate and ammonium recovery as struvite (open-loop recycling).	99.6% phosphate and 98.2% ammonium recovered in laboratory wastewater tests.	Not mentioned. Reagent demand and industrial process performance require evaluation.
[89]	Waste cement-kiln chamotte; ground and alkali-activated.	Geopolymer membrane for wastewater treatment (open-loop recycling).	20 wt.% chamotte incorporated; COD and BOD removal reached 95.74% and 96.80% in laboratory treatment.	Not mentioned. No information was provided regarding the performance and operational lifespan after regeneration.

## Data Availability

No new data were created or analyzed in this study. Data sharing is not applicable to this article.

## References

[1] Ferreira G., Lopez-Sabiron A.M., Aranda J., Mainar-Toledo M.D., Aranda-Uson A. (2015). Environmental Analysis for Identifying Challenges to Recover Used Reinforced Refractories in Industrial Furnaces. J. Clean. Prod..

[2] Seifert S., Dittrich S., Bach J. (2021). Recovery of Raw Materials from Ceramic Waste Materials for the Refractory Industry. Processes.

[3] Xu T., Xu Y., Liao N., Li Y., Nath M. (2021). High-Temperature Chemical Stability of Cr(III) Oxide Refractories in the Presence of Calcium Aluminate Cement. Materials.

[4] Lopez-Perales J.F., Diaz-Tato L., Costilla-Aguilar S.U., Gonzalez-Carranza Y., de Leon J.E.C., Rodriguez-Castellanos E.A. (2025). Fly Ash As a Secondary Raw Material Improving the Sustainable Characteristics of a Conventional Silicoaluminate Refractory Castable. Inorganics.

[5] Horckmans L., Nielsen P., Dierckx P., Ducastel A. (2019). Recycling of refractory bricks used in basic steelmaking: A review. Resour. Conserv. Recycl..

[6] Zeng D.F. (2019). Development trend of China’s refractory industry in the new era. Refractories.

[7] Compilation Group of the Draft Standard for Air Pollutant Emission Standards for the Refractory Industry (2025). Compilation explanation for the Draft Standard for Air Pollutant Emission Standards for the Refractory Industry.

[8] Tianxia Gongchang Industry Research Institute (2026). 2026 China Refractory Materials Industry Deep Research Report (FY2025–2026H1). https://faxiangongchang.com/en/reports/china-refractory-materials-2026.

[9] Salmen S. Closing the Loop: Refractories Recycling. In Mineral Recycling Forum 2022, Dubrovnik, Croatia, 28–30 March 2022; Conference Presentation. https://imformed.com/wp-content/uploads/2022/03/SALMEN-Mineral-Recycling-2022-IMFORMED.pdf.

[10] Feucht F., Moderegger R., Neuhold S., Sedlazeck K.P. (2025). Analysing Material Flows and Final Fate Distribution of Spent Refractories from Steel Casting Ladles and Cement Rotary Kilns in Europe. Resour. Conserv. Recycl..

[11] Dong Z., Wang Z., Zhao Z., Song Y. (2025). The Scrapping Mechanism for the Corundum-Mullite Refractory Material in Rotary Kiln Incinerators. Materials.

[12] Baloyi H., Thethwayo B.M., Mulaba-Bafubiandi A.F., Fosso-Kankeu E., Waanders F., Bulsara H.K.P. (2019). Recycling of ceramic refractory materials. SETWM-19, ACBES-19 & EEHSS-19.

[13] Hemrick J. (2023). Use of Novel Refractory Design and Installation Techniques for Improved Energy Efficiency in Iron and Steel and Other Energy Intensive Industries.

[14] Li S., Wang W., Piao J., Zhou J. (2025). Current Research Status and Development Trends of Recycling and Utilization of Waste Refractory Materials. Cem. Eng..

[15] Munoz I., Soto A., Maza D., Bayon F. (2020). Life Cycle Assessment of Refractory Waste Management in a Spanish Steel Works. Waste Manag..

[16] Mangngi P., Tjaronge M.W., Caronge M.A. (2024). Durability Assessment of Concrete Containing Recycled Coarse Refractory Brick Aggregate. Innov. Infrastruct. Solut..

[17] Pertiwi A.V.D., Caronge M.A., Tjaronge M.W. (2025). Producing Eco-Friendly Concrete Paving Block Using Waste Refractory Brick Aggregates. Int. J. Pavement Res. Technol..

[18] Srivastava R.R., Rajak D.K., Ilyas S., Kim H., Pathak P. (2023). Challenges, Regulations, and Case Studies on Sustainable Management of Industrial Waste. Minerals.

[19] Page M.J., McKenzie J.E., Bossuyt P.M., Boutron I., Hoffmann T.C., Mulrow C.D., Shamseer L., Tetzlaff J.M., Akl E.A., Brennan S.E. (2021). The PRISMA 2020 Statement: An Updated Guideline for Reporting Systematic Reviews. Syst. Rev..

[20] Pei Q., Yan W., Song J., Liu X., Qi J., Li Y., Shi H. (2025). Damage Mechanism of MgO-CaO Refractory for Air Gun Area of AOD Furnace. Bull. Chin. Ceram. Soc..

[21] Ma Z., Li B., Ma G., Du S., Li B., Zhang Y. (2022). Study of thermal degradation performance of refractory concrete under high-temperature jet flow. J. China Univ. Min. Technol..

[22] Gao J., Su W., Gong Y., Chen J., Wang X., Song X., Xu G., Yu G. (2026). Effect of Sodium Species with Different Modes of Occurrence on Corrosion Behavior of High-Chromia Refractory for Entrained-Flow Gasifier Linings. Fuel.

[23] Zhou W., Yan W., Ma S., Schaffoner S., Dai Y., Li Y. (2021). Degradation Mechanisms of Periclase-Magnesium Aluminate Spinel Refractory Bricks Used in the Upper Transition Zone of a Cement Rotary Kiln. Constr. Build. Mater..

[24] Gao J., Su W., Wang X., Song X., Wang J., Yang J., Yu G. (2023). Corrosion and Degradation Mechanisms of High Chromia Refractory Bricks in an Entrained-Flow Gasifier: Experimental and Numerical Analysis. J. Mater. Res. Technol..

[25] Gao J., Liu K., Gong Y., Guo Q., Wang X., Song X., Yu G. (2025). Influence of phosphate additives on the wettability and corrosion resistance of high-chrome refractories against Ca/Na-rich alkaline slag. J. Ind. Eng. Chem..

[26] Liu K., Guo Q., Gong Y., Chen X., Wu T., Wang S., Yu G. (2021). Corrosion of High-Chrome Refractory Materials by High-Sodium Slag in an Entrained-Flow Gasifier. Ceram. Int..

[27] Belgacem S., Galai H., Tiss H. (2016). Qualitative and quantitative investigation of post-mortem cement refractory: The case of magnesia spinel bricks. Ceram. Int..

[28] Cristante Â., Vernilli F. (2021). Comparison of Thermal Shock Damage and Slag Corrosion in Castable Refractories. Interceram-Int. Ceram. Rev..

[29] Liu C., Huang A., Li S., Chen X., Gu H. (2024). Research progress on characterization and evaluation of refractory damage behavior based on machine vision/hearing. J. Iron Steel Res..

[30] Ludwig M., Sniezek E., Jastrzebska I., Prorok R., Sulkowski M., Golawski C., Fischer C., Wojteczko K., Szczerba J. (2021). Recycled Magnesia-Carbon Aggregate As the Component of New Type of MgO-C Refractories. Constr. Build. Mater..

[31] Moritz K., Brachhold N., Kuester F., Dudczig S., Schemmel T., Aneziris C.G. (2023). Studies on the Use of Two Different Magnesia-Carbon Recyclates As Secondary Raw Material for MgO-C Refractories. Open Ceram..

[32] Chen W., Wang B., Liu L., Wang H., Wang X. (2020). Preparation and Slag Erosion Resistance Mechanism of MgAlON Based Composite Refractories Synthesized from Solid Waste. Ceram. Int..

[33] Cheng X., Miao X., Liu L., Zhang Y., Guo M., Cheng F., Zhang M. (2021). Microstructural characteristics and fracture mechanism of in situ pressureless-sintered MgAlON-MgO ceramic from spent MgO-C brick. Mater. Charact..

[34] Cheng X., Liu L., Xiao L., Huo X., Guo M., Zhang M. (2023). Effect of MgO on corrosion mechanism under LF refining slag for MgAlON-MgO composites synthesized from spent MgO-C brick: Wetting behaviour, reaction process and infiltration mechanism. J. Alloys Compd..

[35] Jankovský O., Jiříčková A., Záleská M., Pavlíková M., Pavlík Z., Pivák A., Aneziris C.G., Lauermannová A.-M. (2024). Utilization of Carbon-bonded Magnesia Refractory Waste in MOC-based Composites: Towards CO_2_-neutral Building Materials. Open Ceram..

[36] Capelo-Aviles S., de Oliveira R.T., Stampino I.I.G., Gispert-Guirado F., Casals-Terre A., Giancola S., Galan-Mascaros J.R. (2024). A Thorough Assessment of Mineral Carbonation of Steel Slag and Refractory Waste. J. CO_2_ Util..

[37] Hou H., Hou Z., Liu D., Wang J. (2022). Method for Making High-Quality Refractory Raw Materials and Aluminum-Magnesium-Carbon Bricks by Using the Metamorphic Layer of Waste Aluminum-Magnesium-Carbon Bricks.

[38] Hou H., Hou Z., Liu D., Wang J. (2013). Recovery Process of Used Aluminum-Magnesium-Carbon Bricks and Aluminum-Spinel-Carbon Bricks for Ladles.

[39] Nath M., Xu T., Pandolfelli V.C., Borgese O.H., Liao N., Li Y. (2024). Stabilizing Cr^3+^ in a Mixture of Solid Solution Phases for High-Temperature Applications. J. Eur. Ceram. Soc..

[40] Peng H., Guo J., Lv L., Huang H., Li B. (2021). Recovery of Chromium by Calcium-Roasting, Sodium-Roasting, Acidic Leaching, Alkaline Leaching and Sub-Molten Technology: A Review. Environ. Chem. Lett..

[41] Previato Sardelli J.A., Soares S.E., Loyola L.R., Mendonça G.O., Pereira S.C. (2025). Circular economy in refractory design: Improving magnesia-chromite bricks with recycled raw material. Bulletin.

[42] Silva A.P., Segadães A.M., Lopes R.A. (2017). Castable systems designed with powders reclaimed from dismantled steel induction furnace refractory linings. Ceram. Int..

[43] Matinde E., Msibi S.L. (2019). Effect of reclaimed bauxite on andalusite-based refractory castables for tundish applications. J. S. Afr. Inst. Min. Metall..

[44] Kenzhaliyev B., Biryukova A., Dzhienalyev T., Panichkin A., Imbarova A., Uskenbaeva A., Yusoff A.H. (2024). Assessment of Microsilica as a Raw Material for Obtaining Mullite-Silica Refractories. Processes.

[45] Frade S.B., Gomes G., Correia S.L., Segadaes A.M. (2018). Characterisation of Refractory Ceramic Pressed Body Containing Industrial Waste. Mater. Sci. Forum.

[46] Wang Z., Lyu X., Zhu L., Zhang J., Ma S., Zhang H. (2022). Application of a kind of hydrated magnesia-aluminum spinel bricks. Refract. Lime.

[47] Diaz-Tato L., Lopez-Perales J.F., Contreras J.E., Banda-Munoz F., Suarez-Suarez D., Gonzalez-Carranza Y., Gomez-Rodriguez C., Rodriguez E.A. (2022). Hydration Resistance and Mechano-Physical Properties Improvement of a Magnesia-Dolomite Dense Refractory by Hercynite Spinel. Mater. Chem. Phys..

[48] Xiong N., Deng J. (2021). Improvement of properties of magnesia-calcia refractory by iron oxide nanoparticles. Refract. Lime.

[49] Zheng Y., Luo X., Su Y., Zhao J., Zhang L. (2019). Effect of fused iron-alumina spinel addition on kiln-coating adherence of magnesia-hercynite castables. Refractories.

[50] Kong X., Deng H., Yang L. (2022). Development of high performance magnesia-hercynite bricks for large-scale dry process cement kiln. Refract Lime.

[51] Alfocea-Roig A., Huete-Hernandez S., Garcia-Zubiri I.X., Giro-Paloma J., Formosa J. (2023). Can Tundish Deskulling Waste Be Used As a Magnesium Oxide Source to Develop Magnesium Phosphate Cement?. J. Environ. Chem. Eng..

[52] Kusiorowski R. (2020). MgO-ZrO_2_ refractory ceramics based on recycled magnesia-carbon bricks. Constr. Build. Mater..

[53] Li Z., Zhao H., Zhao P., Zhang H., Yu J. (2022). Utilization of Recycled Corundum As a Refractory Raw Material: Characteristics and Performance Evaluation. Ceram. Int..

[54] de Lourdes Dos Santos Schackow M., Schackow A., Mundstock K.B., Folgueras M.V. (2024). Sustainable Silico-Aluminous Refractory Wastes As an Alternative Raw Material for Hydraulic Binder for Foundry Industry. Clean. Mater..

[55] Dai X., Lian L., Jia X., Qin J., Qian J. (2023). Preparation and Properties of Magnesium Phosphate Cement with Recycled Magnesia from Waste Magnesia Refractory Bricks. J. Build. Eng..

[56] Stadtmüller T.M.J., Storti E., Brachhold N., Lauermannová A.-M., Jankovský O., Schemmel T., Hubálková J., Gehre P., Aneziris C.G. (2023). MgO-C refractories based on refractory recyclates and environmentally friendly binders. Open Ceram..

[57] Lauermannová A.-M., Jankovský O., Stadtmüller T.M.J., Storti E., Aneziris C.G. (2024). Eco-friendly lactose/tannin-based binder in MgO-C refractories produced from MgO-C recyclate. Ceram. Int..

[58] Derensy M., Tonnesen T. (2026). From Metallurgical Waste to Sustainable Refractory Binder: Hydraulic Performance of Vanadium-Bearing Slags. Ceram. Int..

[59] Wang J., Kim H., Go M., Lee D. (2021). Basic Study on the Recycling of a Waste MgO-C Refractory Material As a Flux for EAF Steelmaking. Resour. Recycl..

[60] Liang Z., Liu C.-Y., Du C.-M., Huang F.-X., Sun Y.-L., Jia Y., Wang Y.-F., Ma B.-Y. (2023). Dissolution behavior of spent MgO-C refractory in the CaO-SiO_2_-FeO slag system as a steelmaking flux. Ceram. Int..

[61] Konovalova O.A., Zagorodnov V.Y., Karimov S.M., Amelin A.V., Koverzin A.M. (2015). Use of Scrap of Refractory Articles in the Technology Employed in the Fabrication of Flux. Refract. Ind. Ceram..

[62] Avelar T.D.C., Grillo F.F., Junca E., Coleti J.L., de Oliveira J.R. (2018). Evaluation of Recycled MgO-C Bricks and Dead-Burned Dolomite Fines in Setting Slag Foaming in the Electric Arc Furnace. REM-Int. Eng. J..

[63] Jiao F., Li W., Xue K., Yang C., Qin W. (2019). Recovery of Chromium and Magnesium from Spent Magnesia-Chrome Refractories by Acid Leaching Combined with Alkali Precipitation and Evaporation. Sep. Purif. Technol..

[64] Xue K., Li W., Jiao F., Qin W., Yang C. (2021). Comprehensive Recovery of Valuable Metals From Spent Magnesia-Chrome Refractories by Ferric Chloride-Hydrochloride Leaching. J. Sustain. Metall..

[65] Xue K., Han J., Jiao F., Liu W., Qin W., Cai L., Xu T. (2018). Comprehensive utilization of spent magnesia-chrome refractories with gravity separation followed by flotation. Miner. Eng..

[66] Han J., Jiao F., Liu W., Qin W., Xu T., Xue K., Zhang T. (2016). Innovative Methodology for Comprehensive Utilization of Spent MgO-Cr_2_O_3_ Bricks: Copper Flotation. ACS Sustain. Chem. Eng..

[67] Xue K., Jiao F., Li W., Liu W., Qin W., Yang C. (2022). Using Magnesium Chloride to Volatilize Impurity Metals from Waste Magnesia-Chromium Refractories. JOM.

[68] Hou W., Li H., Li M., Cheng B. (2021). Recycling of spent refractory materials to produce Al-Si master alloys via the aluminum reduction cell. J. Clean. Prod..

[69] Wang X., Li X., Lian L., Jia X., Qian J. (2023). Recycling of Waste Magnesia Refractory Brick Powder in Preparing Magnesium Phosphate Cement Mortar: Hydration Activity, Mechanical Properties and Long-Term Performance. Constr. Build. Mater..

[70] El-Didamony H., Amer A.A., El-Hoseny S. (2016). Recycling of Low-Grade Aluminosilicate Refractory Brick Waste Product in Blended Cement. J. Therm. Anal. Calorim..

[71] Aboutaleb D., Safi B., Chahour K., Belaid A. (2017). Use of Refractory Bricks As Sand Replacement in Self-Compacting Mortar. Cogent Eng..

[72] Ghosh S., Samanta A.K. (2023). Utilization of Recycled Refractory Brick As Fine Aggregate on Various Properties of Sustainable Concrete. Mater. Today Proc..

[73] Malensek N., Ducman V., Mirtic B. (2015). Recycled Granulate Obtained from Waste Alumina-Rich Refractory Powder by the Cold Bonding Process. Ceram. Int..

[74] Arianpour F., Kazemi F., Fard F.G. (2010). Characterization, Microstructure and Corrosion Behavior of Magnesia Refractories Produced from Recycled Refractory Aggregates. Miner. Eng..

[75] Ghosh S., Sinha S., Samanta A.K. (2024). Performance of Recycled Refractory Brick Fine Aggregate on Diverse Properties of Portland Slag Cement Based Heat Resistant Concrete. J. Build. Eng..

[76] Khattab M., Hachemi S., Ajlouni M.F.A. (2021). Recycled Refractory Brick As Aggregate for Eco-friendly Concrete Production. J. Adv. Sci. Eng. Technol..

[77] Khattab M., Hachemi S., Ajlouni M.F.A. (2021). Evaluating the Physical and Mechanical Properties of Concrete Prepared with Recycled Refractory Brick Aggregates after Elevated Temperatures’ Exposure. Constr. Build. Mater..

[78] Sinha S., Ghosh S., Samanta A.K. (2023). Influence of Waste Refractory Brick on the Destructive and Non-Destructive Properties of Sustainable Eco-Friendly Concrete. Mater. Today Proc..

[79] Nematzadeh M., Baradaran-Nasiri A. (2018). Residual Properties of Concrete Containing Recycled Refractory Brick Aggregate at Elevated Temperatures. J. Mater. Civ. Eng..

[80] Nematzadeh M., Dashti J., Ganjavi B. (2018). Optimizing Compressive Behavior of Concrete Containing Fine Recycled Refractory Brick Aggregate Together with Calcium Aluminate Cement and Polyvinyl Alcohol Fibers Exposed to Acidic Environment. Constr. Build. Mater..

[81] Sun S., Qu J., Sun M., Ren X., Gong C., Mu X., Zan W., Zhou Z., Deng C., Ma B. (2024). Preparation and Properties of Lightweight Aggregates from Discarded Al_2_O_3_-ZrO_2_-C Refractories. Materials.

[82] Xue X., Zheng X., Guan B., Liu J., Ding D., Xiong R., Zhao H., Wei F. (2022). Long-term Skid Resistance of High-Friction Surface Treatment of Pavement Using High-Alumina Refractory Waste. Constr. Build. Mater..

[83] Seco A., del Castillo J.M., Perlot C., Marcelino S., Espuelas S. (2023). Recycled Granulates Manufacturing from Spent Refractory Wastes and Magnesium Based Binder. Constr. Build. Mater..

[84] Luza A.L., Simao L., Acordi J., Raupp-Pereira F., Innocentini M.D.M., Montedo O.R.K. (2022). Synthesis of Chemically Bonded Porous Ceramics from MgO-C Refractory Bricks Waste. Ceram. Int..

[85] Li D.-Y., Cho Y.-C., Hsu M.H., Lin Y.-P. (2022). Recovery of Phosphate and Ammonia from Wastewater Via Struvite Precipitation Using Spent Refractory Brick Gravel from Steel Industry. J. Environ. Manag..

[86] Kuzin E.N., Kruchinina N.E. (2022). Brucite-Containing Waste from Refractory Materials Production in Wastewater Purification Processes. Glass Ceram..

[87] Uysal E., Dursun H.N., Guler R., Takmaz U., Kut A., Ceri M., Uysal E.C., Gurmen S. (2024). Waste Refractory Brick Material Added Chitosan/oxidized Pullulan Complex Gel Production and Removal of Heavy Metals from Waste Water. Sci. Rep..

[88] Hammal A. (2025). Preparation of Geopolymer Membranes Using Clay and Some Industrial Wastes to Remove the Organic Load of Pharmaceutical Wastewater. BMC Res. Notes.

[89] Panchal H., Sadasivuni K.K., Shanmugan S., Pandya N. (2020). Performance Analysis of Waste Brick Magnesia As a Storage Material in a Solar Still. Heat Transf..

